# Multimodal and spatially resolved profiling identifies distinct patterns of T cell infiltration in nodal B cell lymphoma entities

**DOI:** 10.1038/s41556-024-01358-2

**Published:** 2024-02-20

**Authors:** Tobias Roider, Marc A. Baertsch, Donnacha Fitzgerald, Harald Vöhringer, Berit J. Brinkmann, Felix Czernilofsky, Mareike Knoll, Laura Llaó-Cid, Anna Mathioudaki, Bianca Faßbender, Maxime Herbon, Tobias Lautwein, Peter-Martin Bruch, Nora Liebers, Christian M. Schürch, Verena Passerini, Marc Seifert, Alexander Brobeil, Gunhild Mechtersheimer, Carsten Müller-Tidow, Oliver Weigert, Martina Seiffert, Garry P. Nolan, Wolfgang Huber, Sascha Dietrich

**Affiliations:** 1 Department of Medicine V, Hematology, Oncology and Rheumatology, University of Heidelberg, Heidelberg, Germany; 2 Molecular Medicine Partnership Unit, Heidelberg, Germany; 3 European Molecular Biology Laboratory, Heidelberg, Germany; 4 Department of Microbiology and Immunology, Stanford University School of Medicine, Stanford, CA USA; 5 Clinical Cooperation Unit Molecular Hematology/Oncology, German Cancer Research Center, Heidelberg, Germany; 6 Division of Molecular Genetics, German Cancer Research Center, Heidelberg, Germany; 7 Molecular Pathology of Lymphoid Neoplasms, Fundació de Recerca Clinic Barcelona-Institut d’Investigacions Biomèdiques August Pi i Sunyer (FRCB-IDIBAPS), Barcelona, Spain; 8 Department of Hematology and Oncology, University Hospital Düsseldorf, Düsseldorf, Germany; 9 Genomics and Transcriptomics Laboratory, University of Düsseldorf, Düsseldorf, Germany; 10 National Center for Tumor Diseases, Heidelberg, Germany; 11 German Cancer Research Center, Heidelberg, Germany; 12 Department of Pathology and Neuropathology, University Hospital and Comprehensive Cancer Center Tübingen, Tübingen, Germany; 13 Department of Medicine III, Laboratory for Experimental Leukemia and Lymphoma Research, Ludwig-Maximilians-University Hospital, Munich, Germany; 14 Department of Pathology, University of Heidelberg, Heidelberg, Germany; 15 German Cancer Consortium, Munich, Germany; 16 Department of Pathology, Stanford University School of Medicine, Stanford, CA USA; 17 Center for Integrated Oncology Aachen-Bonn-Cologne-Düsseldorf (CIO ABCD), Aachen Bonn Cologne Düsseldorf, Germany

**Keywords:** RNA sequencing, Cancer microenvironment, T cells

## Abstract

The redirection of T cells has emerged as an attractive therapeutic principle in B cell non-Hodgkin lymphoma (B-NHL). However, a detailed characterization of lymphoma-infiltrating T cells across B-NHL entities is missing. Here we present an in-depth T cell reference map of nodal B-NHL, based on cellular indexing of transcriptomes and epitopes, T cell receptor sequencing, flow cytometry and multiplexed immunofluorescence applied to 101 lymph nodes from patients with diffuse large B cell, mantle cell, follicular or marginal zone lymphoma, and from healthy controls. This multimodal resource revealed quantitative and spatial aberrations of the T cell microenvironment across and within B-NHL entities. Quantitative differences in PD1^+^ *TCF7*^−^ cytotoxic T cells, T follicular helper cells or IKZF3^+^ regulatory T cells were linked to their clonal expansion. The abundance of PD1^+^ *TCF7*^−^ cytotoxic T cells was associated with poor survival. Our study portrays lymphoma-infiltrating T cells with unprecedented comprehensiveness and provides a unique resource for the investigation of lymphoma biology and prognosis.

## Abstract (web summary)

Roider, Baertsch et al. present a spatially resolved resource map of the T cell infiltration landscape across and within patients with distinct B cell non-Hodgkin lymphoma entities.

## Main

Nodal B cell non-Hodgkin lymphomas (B-NHL) represent a heterogeneous group of indolent and aggressive malignancies. Extensive genetic and transcriptomic profiling has revealed mutational signatures and pathway dependencies, paving the way for molecular therapy^1–8^. However, in recent years, T cell-engaging immunotherapies, such as bispecific antibodies or chimaeric antigen receptor T cells, have emerged among the leading treatment options for patients with refractory and relapsed B-NHL^9–12^. Tailoring these treatment approaches to different B-NHL entities requires systematic investigation of the variety and functions of tumour-infiltrating T cells—analogously to studying the genetic and transcriptomic makeup of tumour cells as a prerequisite for tailoring targeted therapies.

Traditionally, T cell phenotyping studies have been based on immunohistochemistry or flow cytometry^13^. In recent years, single-cell RNA sequencing (scRNA-seq) emerged as a powerful tool and became an integral part of T cell phenotyping efforts^14,15^. We and others have pioneered the investigation of transcriptional heterogeneity of lymph node (LN)-derived T cells in B-NHL, but with the limitation of low sample sizes or having focused only on follicular lymphoma (FL, indolent)^16–18^. Stand-alone scRNA-seq studies additionally face the problem to align gene expression profiles with known T cell subsets that have been defined for decades based on surface epitopes and transcription factors^19^.

In this Resource, we employed cellular indexing of transcriptomes and epitopes by sequencing (CITE-seq), which simultaneously captures transcript and surface epitope abundances at single-cell resolution, and thus enables a multimodal identification of T cell phenotypes^20,21^. Aiming to create a multimodal reference map of LN-derived T cells in nodal B-NHL, we collected more than 100 LN patient samples and included, besides FL, other B-NHL entities with only little or no prior groundwork: diffuse large B cell lymphoma (DLBCL, aggressive), marginal zone lymphoma (MZL, indolent) and mantle cell lymphoma (MCL, mixed). We identified and quantitated fine-grained T cell subsets and determined their clonality using full-length single-cell T cell receptor (scTCR) sequencing. We further assessed the ability of multicolour flow cytometry and multiplexed immunofluorescence to reproduce these T cell subsets and to localize them within the tumour microenvironment.

## Results

### Study and sample overview

**Extended Data Fig. 1:**
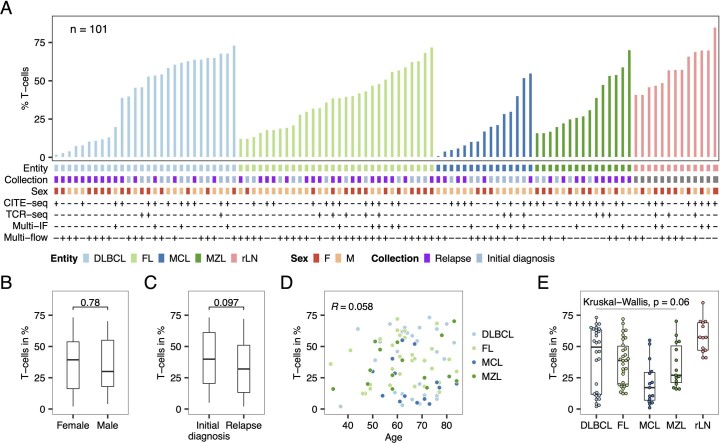
Basic patient characteristics. **a**) Overview showing entity, pre-treatment status, sex, assay availability and the overall T-cell proportion of a total 101 lymph node samples used in this study. **b-e**) Box or scatter plots illustrating the associations of patient characteristics in n = 101 biologically independent patient samples with the overall T-cell proportion determined by flow cytometry. P values and/or correlation coefficients were calculated using the two-sided Wilcoxon-test (B, C), Pearson’s linear correlation (D) or Kruskal-Wallis-test (E). Box plots: centre line, median; box limits, first and third quartile; whiskers, 1.5 x interquartile range. Source data

We collected 101 LN samples (Extended Data Fig. 1a and Supplementary Table 1) from patients with DLBCL (*n* = 28), FL (*n* = 30), MZL (*n* = 15), MCL (*n* = 15), or from patients without evidence of malignancy (tumour-free/reactive LN (rLN), *n* = 13). LN samples from 38 patients were collected at initial diagnosis, while 50 LN samples were collected from patients who had received one or more prior lines of systemic treatment (Extended Data Fig. 1a). To minimize potential effects on T cell infiltration patterns, relapse samples were collected at least 3 months after cessation of the preceding treatment. T cell proportions of malignant LN samples determined by flow cytometry showed a broad variation (Extended Data Fig. 1a) but were not significantly associated with pre-treatment status, sex, age or B-NHL entity (Extended Data Fig. 1b–e).

### Fourteen multimodally defined T cell subsets

**Fig. 1:**
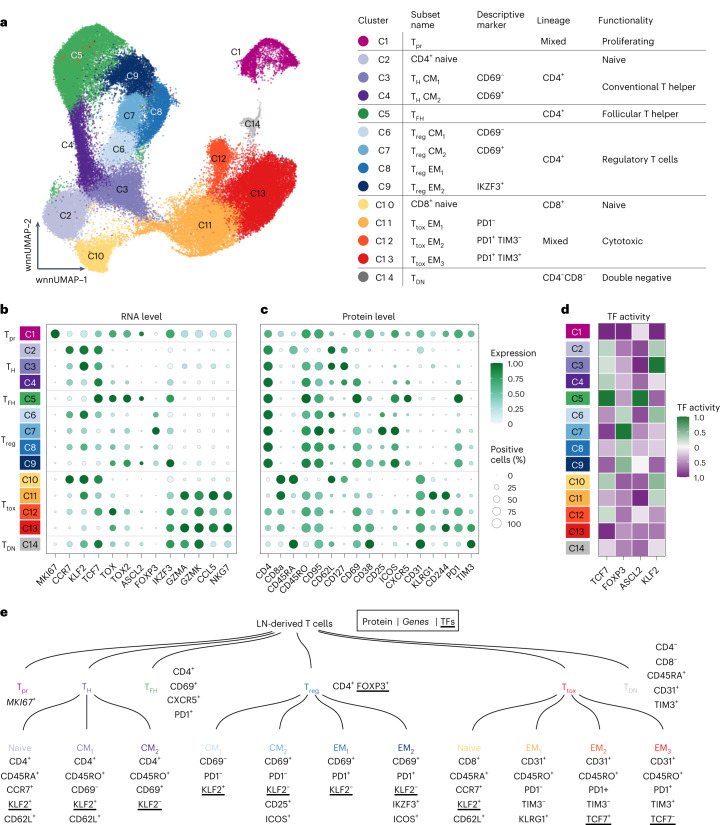
LN-derived T cells can be divided into 14 multimodally defined subsets. **a**, CITE-seq data from 51 primary LN patient samples were integrated and jointly visualized using UMAP. Cells were coloured with respect to their cluster on the basis of a shared nearest neighbour-based algorithm. The adjacent table summarizes all clusters including used subset names, lineages and functionality. **b**,**c**, Dot plot showing the expression of important marker genes and proteins to identify all T cell subsets. Size and colour of the dots indicate the percentage of positive cells and scaled gene/protein expression, respectively. Values were scaled between 0 and 1. **d**, Heatmap showing the inferred activity of selected transcription factors (TFs), as indicated. *y* axis is identical to **b** and **c**. Values were scaled between −1 and 1. **e**, Dendrogram summarizing the 14 multimodally defined T cell subsets including most important and interpretable marker genes, proteins and transcription factors. Source data

We used CITE-seq to profile T cells from 51 LN patient samples. Surface proteins were detected using 70 oligonucleotide-tagged antibodies (Supplementary Table 2). After quality control and in silico sorting, we obtained data for 74,112 CD3^+^ T cells with a median of 1,190 T cells per patient sample. Unsupervised clustering based on a weighted combination of transcriptome and epitope similarities (weighted nearest neighbour)^22^ grouped the T cells into proliferating (T_pr_), conventional helper (T_H_) and follicular helper (T_FH_), regulatory (T_reg_), cytotoxic (T_tox_) and double-negative T cells (T_DN_, Fig. 1a). Differentially expressed genes and proteins (Fig. 1b,c) were associated with lineage (CD4 and CD8), functional specialization (for example, *FOXP3* and *ASCL2*), cytotoxicity (for example, *GZMA* and *GZMK*) or proliferation (for example, *MKI67*). These groups could be further partitioned into CD4^+^ and CD8^+^ naive T cells, central memory (CM_1_ and CM_2_) T_H_ cells, central memory (CM_1_ and CM_2_) and effector memory (EM_1_ and EM_2_) T_reg_ cells, and effector memory (EM_1_, EM_2_ and EM_3_) T_tox_ cells. At this level of granularity, differentially expressed markers (Fig. 1b,c) were linked to differentiation (for example, CD45RA, CD45RO and CD62L), homing and migration (for example, *KLF2* and *CCR7*), activation (for example, CD69, CD38 and CD278) and inhibition (for example, PD1, TIM3 and LAG3). This high-granularity classification was supported by a gene regulatory network analysis^23^, which highlighted differential activities of specific transcription factors (for example, KLF2 (ref. ^24^), TCF7 (ref. ^25^), FOXP3 (ref. ^26^) and ASCL2 (ref. ^27^), Fig. 1d). On this basis, we compiled profiles of the most discriminating and biologically interpretable surface proteins, genes and transcription factors (Fig. 1e). Further extended profiles are provided in Supplementary Table 3.

### Cytometry reproduces multimodally defined T cell subsets

**Fig. 2:**
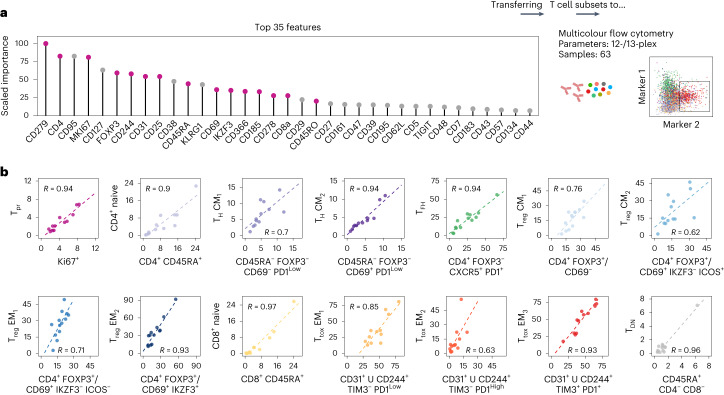
Multicolour flow cytometry reproduces multimodally defined T cell subsets. **a**, Most important features to distinguish multimodally defined T cell subsets using a gradient boosting classifier. Only features that are routinely accessible by flow cytometry were considered for the model. **b**, Percentages of all T cell subsets determined by flow cytometry (*x* axis) and CITE-seq (*y* axis) were correlated for *n* = 13 biologically independent samples. *x* axis title indicates the applied gating strategy. The symbol ‘U’ indicates merging of two populations. Pearson’s correlation coefficient is given for each panel (*R*). Source data

**Extended Data Fig. 2:**
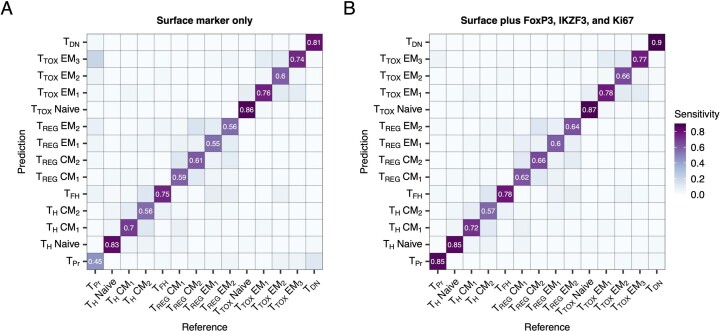
Confusion tables comparing flow cytometry and CITE-seq. **a-b**) Gradient boosting classifiers were trained and tested to evaluate whether T-cell subsets identified by CITE-seq can be predicted on basis of surface markers (A) or surface markers and differentially expressed intracellular markers accessible by flow cytometry (B, FoxP3, IKZF3, Ki67). Shown is the proportion of correctly predicted cells per T-cell subset across the complete CITE-seq data set (n = 51 bvles). T_Pr_: Proliferating T-cells. T_H_: Helper T-cells. T_FH_: Follicular helper T-cells. T_REG_: Regulatory T-cells. T_TOX_: Cytotoxic T-cells. T_DN_: Double negative T-cells. CM: Central memory. EM: Effector memory. Source data

Next, we built gradient boosting classifiers^28^ to identify the most discriminatory surface markers between multimodally defined subsets (Fig. 2a). While this yielded accurate results for most T cell subsets (Extended Data Fig. 2a), the distinction among T_reg_ cells and the detection of T_pr_ cells could be improved by additional intracellular markers (Ki67, FOXP3 and IKZF3; Extended Data Fig. 2b). After removal of redundant (for example, CD95 and CD127) and less important markers, we thus compiled a 12- and 13-plex flow cytometry panel (Supplementary Table 4) and established gating strategies supported by the hypergate algorithm^29^, which enabled classification of all 14 multimodally defined T cell subsets (Supplementary Figs. 1 and 2). We correlated the subset proportions determined by CITE-seq and flow cytometry in a total of 13 LN samples and observed a median Pearson coefficient of 0.92 across all subsets (Fig. 2b). We then applied these panels to an independent cohort of 50 LN samples, which was then used for further quantitative analysis of T cell infiltration patterns.

### Quantitative patterns of T cell infiltration in nodal B-NHL

**Fig. 3:**
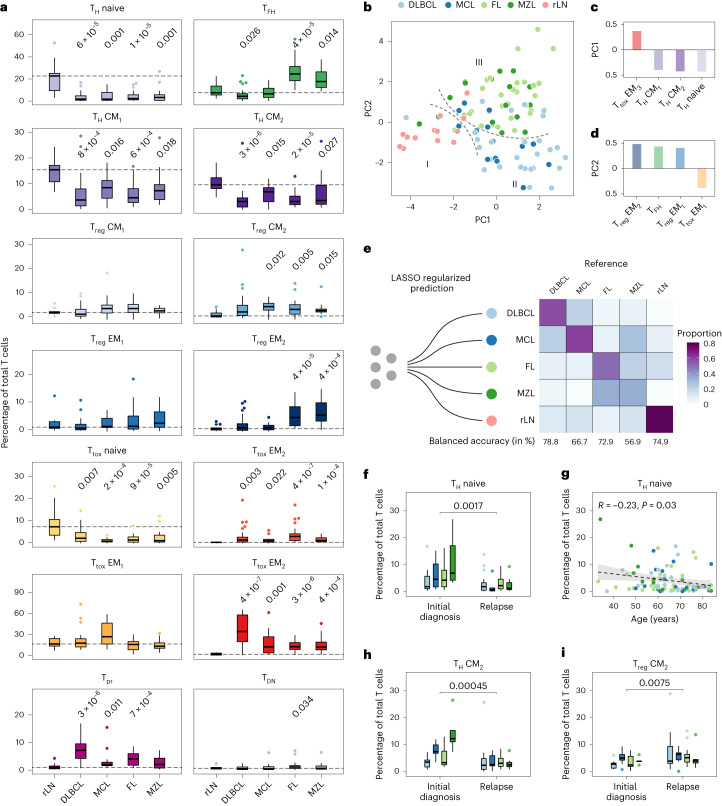
Nodal B-NHL entities have characteristic quantitative patterns of T cell infiltration. **a**, T cell subset proportions determined by CITE-seq or flow cytometry are illustrated in box plots (*n* = 101 biologically independent patient samples). Outliers are shown as individual dots. Each entity and subset were tested versus tumour-free samples (rLN) using a two-sided Wilcoxon test. *P* values were corrected for multiple testing using the Benjamini–Hochberg procedure. Only *P* values ≤0.05 are shown. Dashed lines indicate the median of rLN. **b**–**d**, PCA based on the subset and overall T cell proportions (**b**) including the top four loadings of PC1 (**c**) and PC2 (**d**) are shown. Dashed lines (**b**) highlight three groups (I–III) of samples. **e**, Confusion matrix based on a LASSO-regularized multinomial logistic regression model and estimated classification accuracy using leaving-one-out cross-validation based on subset and overall T cell proportions. **f**–**i**, Patient characteristics were evaluated in a multivariate model regarding their effect on the proportions of all 14 T cell subsets. Shown are the four most significant associations. *P* values and/or correlation coefficients were calculated using a two-sided Wilcoxon test (**f**, **h** and **i**) or Pearson’s linear correlation (**g**). Box plots or dots are coloured by entity as in **b** and **e**. The error band in **g** indicates the 95% confidence interval. Box plots: centre line, median; box limits, first and third quartile; whiskers, 1.5× interquartile range. Source data

**Extended Data Fig. 3:**
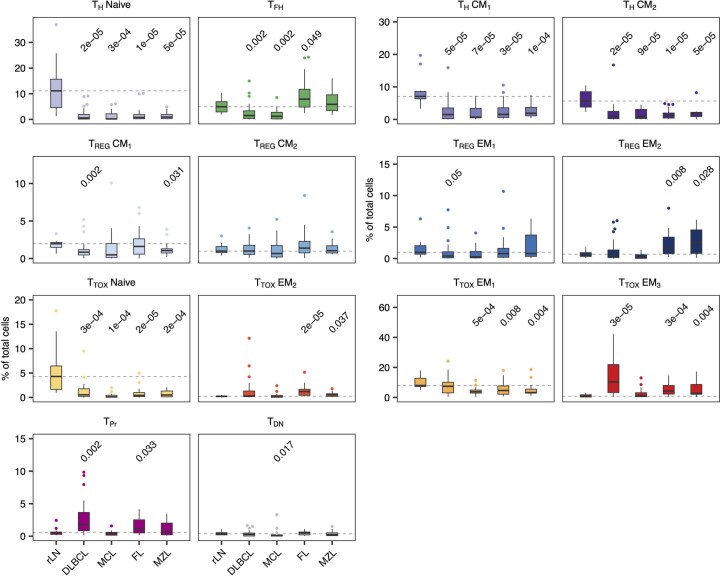
Overall frequencies of T-cell subsets determined by CITE-seq or flow cytometry. Overall frequencies of T-cell subsets determined by CITE-seq or flow cytometry are illustrated in box plots (n = 101). Outliers are shown as individual dots. Each entity and subset were tested versus rLN using a two-sided Wilcoxon-test. P values were corrected for multiple testing using the Benjamini-Hochberg procedure. Only p values ≤ 0.05 are shown. Dashed lines indicate the median of rLN. rLN: Tumor-free lymph nodes. T_Pr_: Proliferating T-cells. T_H_: Helper T-cells. Box plots: centre line, median; box limits, first and third quartile; whiskers, 1.5 x interquartile range. T_FH_: Follicular helper T-cells. T_REG_: Regulatory T-cells. T_TOX_: Cytotoxic T-cells. T_DN_: Double negative T-cells. CM: Central memory. EM: Effector memory. Source data

We combined CITE-seq and flow cytometry data, determined the proportion of each subset per sample, and compared each B-NHL entity with tumour-free LN samples (Fig. 3a). B-NHL were characterized by a lack of CD69^−^ CM_1_ and CD69^+^ CM_2_ T_H_ cells, and CD4^+^/CD8^+^ naive T cells (Fig. 3a). Conversely, PD1^+^ TIM3^−^ T_tox_ EM_2_ cells and PD1^+^ TIM3^+^ EM_3_ T_tox_ cells, T_pr_ cells and CD69^+^ T_reg_ CM_2_ cells were significantly enriched in B-NHL (Fig. 3a). FL and MZL were additionally characterized by significant enrichment of T_FH_ and IKZF3^+^ T_reg_ EM_2_ cells (Fig. 3a). We also noted a significant increase of CD69^+^ T_reg_ CM_2_ cells in MCL, FL and MZL, whereas T_FH_ cells were depleted in DLBCL (Fig. 3a). We observed similar results when using (absolute) subset frequencies instead of (relative) subset proportions (Extended Data Fig. 3). To gain a broader overview of these differences across all B-NHL entities, we used principal component analysis (PCA) on the table of subset proportions and the overall T cell frequency (Fig. 3b). Based on the first two principal components (PCs), we identified three major groups (I–III) represented by tumour-free (I), DLBCL and MCL (II), and FL and MZL LNs (III, Fig. 3b). PC1 (Fig. 3c) and PC2 (Fig. 3d) had high loadings on the characteristic T cell subsets highlighted in Fig. 3a: CD4^+^ and CD8^+^ naive T cells, and PD1^+^ TIM3^+^ T_tox_ EM_3_ cells (PC1), and T_FH_ and IKZF3^+^ T_reg_ EM_2_ cells (PC2).

**Extended Data Fig. 4:**
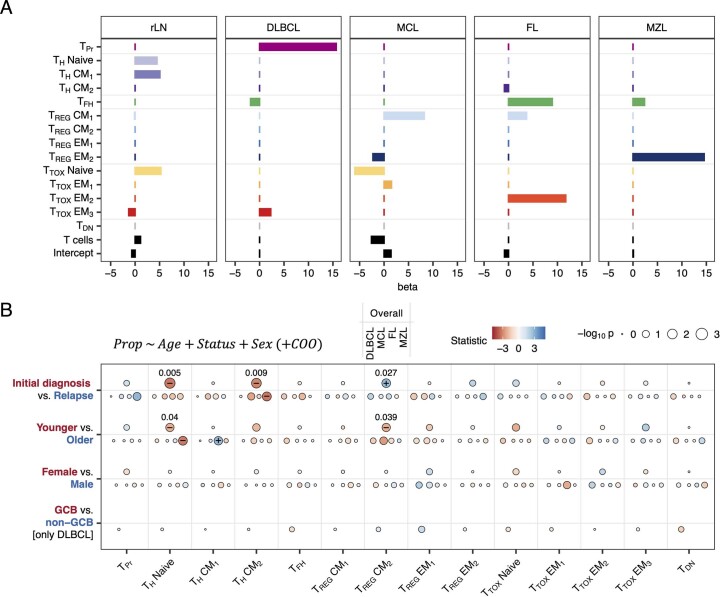
Multinomial and multivariate models to explain varying T-cell subset proportions. **a**) Beta coefficients derived from a LASSO-regularized multinomial regression model predicting rLN, MZL, FL, MCL, or DLBCL based on the subset and overall T-cell proportions. **b**) Multivariate linear models were fit using sex, age, treatment status, COO (only DLBCL) as covariates, and the proportion of each T-cell subset as dependent variable in a two-sided fashion. Models were fit for the total dataset and for each entity separately, as indicated. Diameter and color of the dots indicate –log10 p value and size of the coefficient, respectively. P values were corrected using the Benjamini-Hochberg procedure and significant p values are indicated by +/−. A-B) Analysis is based on n = 101 biologically independent samples. T_Pr_: Proliferating T-cells. T_H_: Helper T-cells. T_FH_: Follicular helper T-cells. T_REG_: Regulatory T-cells. T_TOX_: Cytotoxic T-cells. T_DN_: Double negative T-cells. CM: Central memory. EM: Effector memory. COO: Cell-of-origin. Source data

To further explore to what extent T cell composition and frequency is distinctive for different B-NHL entities and tumour-free LN, we built classifiers using LASSO-regularized multinomial logistic regression and estimated classification accuracy using nested leave-one-out cross-validation (Fig. 3e and Extended Data Fig. 4a). Accuracy was best for distinguishing between tumour-free and malignant LNs (balanced accuracy of 74.5%); moreover, DLBCL and MCL could be differentiated with similar accuracy (Fig. 3e). A third, well-distinguishable group was formed by FL and MZL (Fig. 3e). These results indicate that different entities have distinct patterns of T cell infiltration (Extended Data Fig. 4a), although—based on our current data—classification does not provide diagnostic accuracy.

To explore the potential role of patient-inherent characteristics, we fit multivariate linear models using sex, age, treatment status and cell-of-origin (only DLBCL) as covariates, and the proportion of each T cell subset as dependent variable (Extended Data Fig. 4b). We found that pre-treatment (Fig. 3f, *P* < 0.001) and higher age (Fig. 3g, *P* = 0.04) were associated with a lower proportion of naive CD4^+^ T cells. Pre-treatment was also linked to a lower proportion of CD69^+^ T_H_ CM_2_ cells (Fig. 3h, *P* < 0.001) and a higher proportion of CD69^+^ T_reg_ CM_2_ cells (Fig. 3i, *P* < 0.001), while we observed no statistically significant effect on the T cell composition for sex and cell-of-origin (Extended Data Fig. 4b). Larger sample sizes might be necessary to detect less strong associations, but overall, patient characteristics had only a moderate effect on the T cell composition compared with entity-specific differences.

### Entity-specific clonality of CD4^+^ and CD8^+^ T cell subsets

**Fig. 4:**
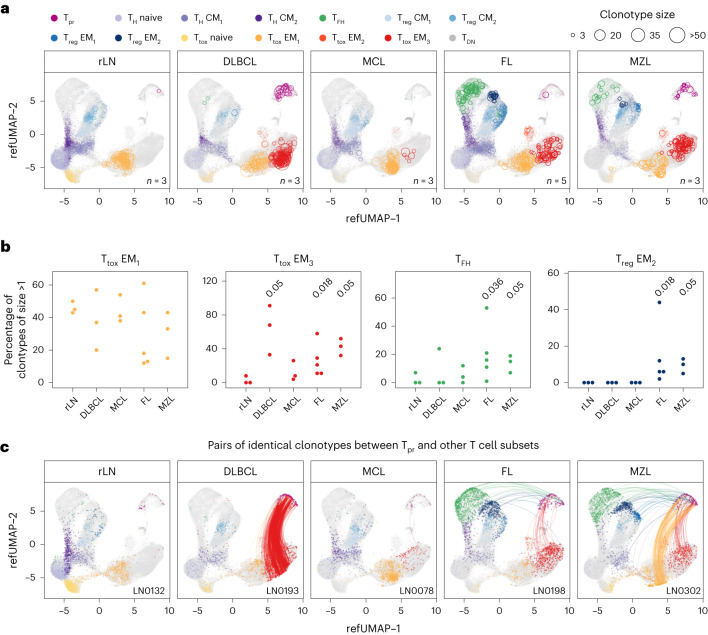
Entity-specific T cell compositions result from differential clonal expansion of CD4^+^ and CD8^+^ T cell subsets. **a**,**b**, 5′ scRNA alongside full-length TCR repertoire data were mapped to the CITE-seq reference dataset. In grey, all cells with 5′ scRNA data are shown, whereas coloured cells belong to samples derived from specific entities (**a**) or samples (**b**), as indicated. The number of biologically independent samples is indicated in each panel. In **a**, circles represent the number of cells with identical TCR clonotype within the same subpopulation. To avoid overplotting, a maximum of 30 circles per sample and T cell subset is shown. In **b**, the percentage of clonally expanded T cells per patient and T cell subset, as indicated, was quantified and compared with tumour-free samples. Each entity and subset were tested versus tumour-free samples (rLN) using a one-sided Wilcoxon test. Only *P* values ≤0.05 are shown. **c**, Mapped cells from five representative samples. Lines connect all proliferating cells with any other cell given that both have identical TCR clonotypes. Source data

**Extended Data Fig. 5:**
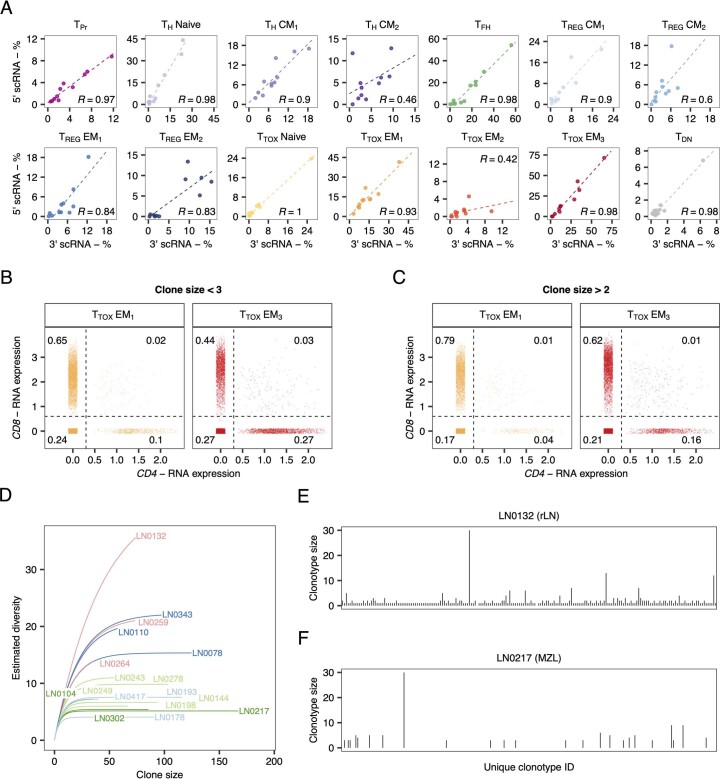
5′ scRNA alongside full-length TCR sequencing and TCR diversity. 5′ scRNA alongside full-length TCR repertoire data from n = 17 biologically independent lymph node samples were mapped to the CITE-seq reference data. **a**) T-cell subset proportions determined by 5′ scRNA or CITE-seq reference data were correlated across 17 patient samples and 14 multimodally defined T-cell subsets. R values represent Pearson’s linear correlation coefficients. **b-c**) CD4 and CD8 gene expression of T_TOX_ EM_1_ and T_TOX_ EM_3_ cells with clone size samller (B) or greater / equal 3 (C). Dots are jittered in x and y direction. Numbers indicate proportions of CD4^+^, CD8^+^, CD4^+^ CD8^+^, or CD4^−^CD8^−^ T-cells. **d**) TCR diversity was esimated for each LN patient sample using a rarefraction analysis (D). **e-f**) TCR diversity is exemplarily illustrated showing the 10 % most abundant clonotypes per sample. T_Pr_: Proliferating T-cells. T_H_: Helper T-cells. T_FH_: Follicular helper T-cells. T_REG_: Regulatory T-cells. T_TOX_: Cytotoxic T-cells. T_DN_: Double negative T-cells. CM: Central memory. EM: Effector memory. TCR: T-cell receptor. Source data

To investigate whether the enrichment of T cell subsets results from their clonal expansion, we performed full-length scTCR sequencing alongside 5′ scRNA-seq in a representative subset of 17 patient samples (Extended Data Fig. 1a and Supplementary Table 1). After quality control, 5′ scRNA and full-length TCR data were available for 66,896 T cells with a median of 3,045 cells per patient sample. We mapped the 5′ scRNA onto the CITE-seq reference data above, indicating high consistency of the inferred subset proportions between both modalities with a median correlation coefficient of *R* = 0.92 (Extended Data Fig. 5a). Then, we compiled the scTCR data to clonotypes on the basis of their complementarity-determining regions^30^ and projected them onto the reference uniform manifold approximation and projection (UMAP; Fig. 4a). Clonally expanded T_tox_ EM_1_ cells were present across all entities and in tumour-free LNs (Fig. 4a), while clonally expanded PD1^+^ TIM3^+^ T_tox_ EM_3_ cells were limited to DLBCL, FL and MZL (Fig. 4a). Clonality of T_tox_ cells was not restricted to *CD8*^+^ but included also *CD4*^*+*^ T_tox_ cells (Extended Data Fig. 5b,c). In addition, T_FH_ and IKZF3^+^ T_reg_ EM_2_ cells were clonally expanded exclusively in FL and MZL (Fig. 4a). Consequently, the TCR diversity was substantially reduced in DLBCL, FL and MZL samples compared with tumour-free and MCL samples (Extended Data Fig. 5d–f). To further support our findings, we quantified the proportion of clonally expanded T cells per subset and compared each entity with tumour-free samples. While T_tox_ EM_1_ cells were clonally expanded to a similar extent across all sample types, T_tox_ EM_3_ cells, T_FH_ cells and T_reg_ EM_2_ cells had significantly enriched proportions of clonally expanded T cells only in entities specified above (Fig. 4b). We further used scTCR data to track the original identity of T_pr_ cells, that is at the time of sample collection temporarily masked by an S or G_2_M phase-dependent gene expression signature^31^. Apart from MCL and tumour-free LN, which both harboured very low proportions of T_pr_ cells (Fig. 4a,c), we identified groups of shared clonotypes in DLBCL, FL and MZL predominantly between T_pr_ cells and PD1^+^ TIM3^+^ T_tox_ EM_3_ cells (Fig. 4c). To a lower extent, TCR clonotypes were also shared between T_pr_ cells and T_FH_ cells, and between T_pr_ cells and IKZF3^+^ T_reg_ EM_2_ cells in FL and MZL (Fig. 4c). Overall, this analysis highlights that an altered T cell microenvironment results from active proliferation and differential expansion of specific T cell subsets.

### PD1^+^ TCF7^−^ T_tox_ cells converge into exhausted T cells

**Fig. 5:**
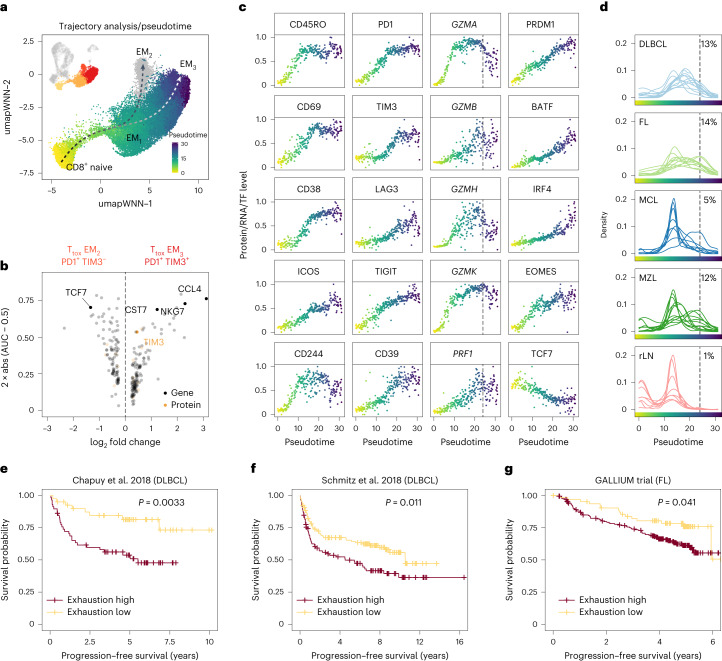
PD1^+^ TCF7^−^ cytotoxic T cells converge into terminally exhausted T cells with variable proportions within and across entities. **a**, Combined trajectory and pseudotime analysis were performed using CITE-seq expression profiles of T_tox_ cells starting from naive CD8^+^ T cells. Arrows illustrate trajectories, while cells are coloured by pseudotime. **b**, Volcano plot illustrating differentially expressed genes and proteins between PD1^+^ TIM3^+^ T_tox_ EM_3_ cells and PD1^+^ TIM3^−^ T_tox_ EM_2_ cells. **c**, Protein expression (first and second column), gene expression (third column) or inferred transcription factor activity (fourth column) are illustrated along binned pseudotime, as shown in **a**. Values were scaled between 0 and 1. Dashed lines indicate threshold when T cells were considered terminally exhausted. **d**, Shown is the density of cells for each single patient along pseudotime. Number indicates median percentage of terminally exhausted T cells across all LN patient samples for each entity. **e**–**g**, Bulk RNA-seq data from patients with DLBCL (**e** and **f**) and FL (**g**) were deconvoluted on the basis of a gene expression signature of terminally exhausted T cells. Kaplan–Meier plots with *P* values of corresponding log-rank test. AUC, area under the curve. Source data

To unveil the process of T cell exhaustion across B-NHL entities in detail, we performed a trajectory analysis^32^ of T_tox_ cells based on the CITE-seq data. We identified two paths: (I) one from naive to PD1^+^ TIM3^−^ T_tox_ EM_2_ cells, and (II) another one from naive to PD1^+^ TIM3^+^ T_tox_ EM_3_ cells (Fig. 5a). Apart from TIM3, both expression (Fig. 5b) and inferred transcription factor activity^23^ of *TCF7* was an important discriminator between both trajectories (Fig. 1d). TCF1 (encoded by *TCF7*) is a hallmark of stemness and longevity and its presence (trajectory I) or absence (trajectory II) indicates maintained or impaired self-renewal capacity, respectively^33^, thereby suggesting that only trajectory II converges into terminally exhausted T cells.

**Extended Data Fig. 6:**
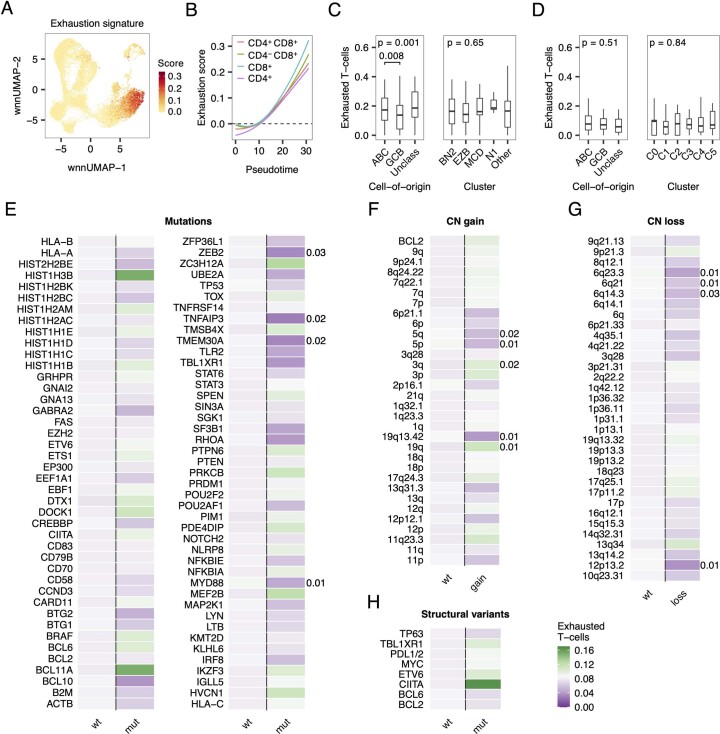
T-cell exhaustion associated with genetic features. **a**) An exhaustion score was calculated based on a transcriptional signature of terminally exhaustion T-cells (see Method section for details) and then projected onto the reference UMAP plot. **b**) Locally estimated scatterplot smoothing (loess) of pseudotime versus exhaustion score (as shown in panel A) separately for CD4^+^, CD8^+^, CD4^+^CD8^+^, and CD4^−^CD8^−^ T_TOX_ cells. **c-h**) Bulk RNA-seq data from DLBCL patients from the Schmitz et al. cohort (C, n = 190) or the Chapuy et al. cohort (D-H, n = 137) were deconvoluted based on a gene expression signature of terminally exhausted T-cells. Box plots (C-D) show associations between T-cell exhaustion and cell-of-origin or genetic subtypes. Conditions were tested for significance using the Kruskal-Wallis-test. Wilcoxon-test was used as post-hoc test (C). Heatmaps (E-H) illustrate the mean estimated proportion of exhausted T-cells for somatic variants (E), amplifications (F), deletions (G), or structural variants (H), as indicated, based on deconvoluation of the bulk RNA-seq of the Chapuy et al. cohort (n = 137). Conditions were tested for significance using the two-sided Wilcoxon-test and corrected for multiple testing using the Benjamini-Hochberg procedure. After correction, all p values were > 0.05. To highlight the strongest differences, uncorrected p values ≤ 0.05 are shown to the right of each panel. Box plots: centre line, median; box limits, first and third quartile; whiskers, 1.5 x interquartile range. ABC: Activated B-cell subtype. GCB: Germinal center B-cell subtype. CN: Copy number. Source data

To further resolve gradual changes along trajectory II, we applied pseudotime analysis^32^ and ranked the cells starting from naive T_tox_ cells (Fig. 5a). We found that pseudotime was strongly linked to a continuously increasing expression of both differentiation and activation markers, such as CD45RO, CD69, CD38 and ICOS, and inhibitory molecules, such as PD1, TIM3, LAG3, TIGIT and CD39 (Fig. 5c). Cells with the highest levels of inhibitory receptors had reduced RNA expression levels of effector molecules, particularly of *GZMA* and *GZMH*, and reduced protein levels of CD244 (ref. ^34^) (Fig. 5c). Likewise, the inferred activity^23^ of the transcription factors PRDM1, BATF, IRF4 and EOMES, which have previously been associated with T cell exhaustion^35–38^, were strongest in T_tox_ cells at the end of the trajectory (highest pseudotime), whereas the inferred transcription factor activity of *TCF7* was lowest in these cells (Fig. 5c). On this basis, we established a signature profile (Supplementary Table 5), to facilitate the identification of terminally exhausted T cells in scRNA-seq data (Extended Data Fig. 6a). While the initial trajectory and pseudotime analysis was performed on complete T_tox_ cells, scoring gave similarly high values when applied separately to CD4^+^ or CD8^+^ T_tox_ cells (Extended Data Fig. 6b).

Plotting the proportion of T_tox_ cells by sample and pseudotime revealed that terminally exhausted T cells were most abundant in DLBCL and FL, most variable in MZL, and lowest in MCL (Fig. 5d). Clonality analysis based on scTCR data supported this finding by demonstrating that the expansion of PD1^+^ TIM3^+^ T_tox_ EM_3_ cells was a key feature of the tumour microenvironment of DLBCL, FL and MZL, while MCL and tumour-free LNs were predominantly characterized by clonal PD1^−^ T_tox_ EM_1_ cells (Fig. 4a).

### T cell exhaustion is linked to adverse prognosis in B-NHL

To investigate if T cell exhaustion is associated with clinical outcome in B-NHL, we extracted a transcriptional signature from terminally exhausted T cells of our data and applied digital cytometry^39^ to bulk RNA data from two large independent retrospective DLBCL cohorts^2,4^. We found that a higher proportion of terminally exhausted T_tox_ cells was associated with inferior progression-free survival in both cohorts (Fig. 5e, *P* = 0.003, Fig. 5f, *P* = 0.011). Moreover, the cohort from Schmitz et al.^4^ harboured higher proportions of exhausted T cells in ABC- than GCB-subtype DLBCL (Extended Data Fig. 6c), which is in line with a recent flow cytometry-based study^40^. However, there was no difference between ABC- and GCB-subtype DLBCL in our data (Extended Data Fig. 4b) or in the cohort from Chapuy et al.^2^ (Extended Data Fig. 6d). Neither of the genetic subtypes defined in these cohorts were associated with the proportion of exhausted T_tox_ cells (Extended Data Fig. 6c,d). We also evaluated individual somatic mutations (for example, *MYD88*), amplifications (for example, *BCL2*), deletions (for example, 17p) and structural variants (for example, *BCL6*). After correction for multiple testing, none of the genetic aberrations was associated with the proportion of exhausted T_tox_ cells (Extended Data Fig. 6e–h). We performed a similar analysis using bulk RNA-seq data of a prospective FL cohort of the GALLIUM trial (NCT01332968)^41,42^, and again a higher proportion of exhausted T cells was associated with inferior survival (Fig. 5g, *P* = 0.04).

### IKZF3^+^ T_reg_ EM_2_ cells are clonally related to T_FH_ cells

**Fig. 6:**
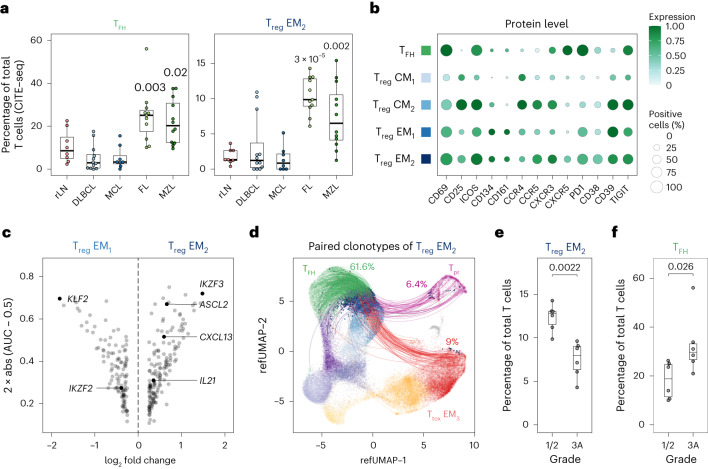
IKZF3^+^ T_reg_ EM_2_ cells are clonally related to T_FH_ cells and associated with grading of FL. **a**, Proportions of T_reg_ EM_2_ cells and T_FH_ cells determined by CITE-seq are illustrated as box plots (*n* = 51 biologically independent patient samples). All entities were tested for significance using a two-sided Wilcoxon test with rLN as reference. Only *P* values ≤0.05 are shown. **b**, Dot plot showing the expression of important phenotypic proteins. Size and colour of the dots indicate the percentage of positive cells and scaled protein expression, respectively. Values were scaled between 0 and 1. **c**, Volcano plot illustrating differentially expressed genes between T_reg_ EM_2_ cells and EM_1_ cells. **d**, 5′ scRNA alongside full-length TCR repertoire data were mapped to the CITE-seq reference data. Lines connect all T_reg_ EM_2_ cells with any other cell given that both T cells have the same TCR clonotype. Percentages indicate shares of overlapping clonotypes for T_FH_ cells, T_pr_ cells and T_tox_ cells. Analysis is based on *n* = 17 biologically independent patient samples. **e**,**f**, Proportions of T_reg_ EM_2_ cells (**e**) and T_FH_ cells (**f**) determined by CITE-seq are shown in dependence of tumour grading in FL (1/2 versus 3A). Differences were tested for significance using a two-sided Wilcoxon test. Shown are *n* = 12 biologically independent patient samples. Box plots: centre line, median; box limits, first and third quartile; whiskers, 1.5× interquartile range. Source data

**Extended Data Fig. 7:**
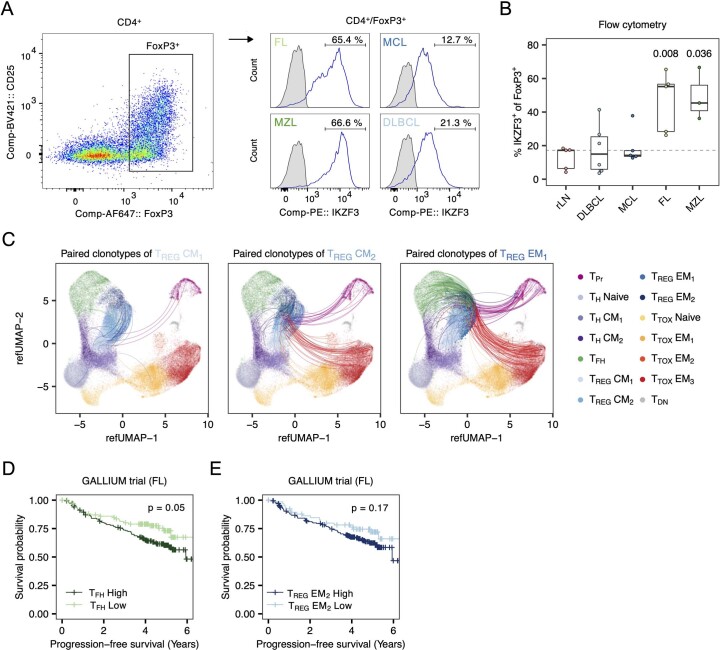
T_REG_ EM_2_ express IKZF3 at protein level and have no impact on overall survival in FL. **a**) Representative pseudocolor and density plots showing the IKZF3 (Aiolos) protein expression in CD4^+^ FoxP3^+^ cells determined by flow cytometry. Numbers indicate percentage of positive cells based on gates as inidicated. Grey shaded histogramm represent fluorescence-minus-one control. **b**) IKZF3 protein expression in FoxP3^+^ T-cells determined by flow cytometry in n = 24 biologically independent B-cell lymphoma patient samples. Each entity was tested versus tumour-free samples (rLN) using the two-sided Wilcoxon-test. Only p values ≤ 0.05 are shown. **c**) 5′ scRNA alongside full-length TCR repertoire data from 11 biologically independent samples were mapped to the CITE-seq reference data. Lines connect all T_REG_ CM_1_, T_REG_ CM_2_ or T_REG_ EM_1_ cells with any other cell given that both T-cells have the same TCR clonotype. **d-e**) Bulk RNA-seq data from patients with FL were deconvoluted based on gene expression signature of T_REG_ EM_2_ (D) or T_FH_ cells (E). Kaplan-Meier plots with p values of corresponding log-rank test. Box plots: centre line, median; box limits, first and third quartile; whiskers, 1.5 x interquartile range. TCR: T-cell receptor. T_Pr_: Proliferating T-cells. T_H_: Helper T-cells. T_FH_: Follicular helper T-cells. T_REG_: Regulatory T-cells. T_TOX_: Cytotoxic T-cells. T_DN_: Double negative T-cells. CM: Central memory. EM: Effector memory. Source data

In FL and MZL, not only T_tox_ cells but also T_FH_ and T_reg_ EM_2_ cells were clonally expanded (Fig. 4a) and significantly enriched (Fig. 6a). While T_FH_ cells are well characterized and known to promote the growth of malignant B cells in FL^43^, the role of LN-derived T_reg_ cells in nodal B-NHL has not been investigated systematically. Aiming to characterize T_reg_ EM_2_ cells, we compared protein and gene expression profiles of T_reg_ EM_2_ cells and T_reg_ EM_1_ cells, and found that T_reg_ EM_2_ cells were characterized by high protein levels of CD69, ICOS, CD38, PD1 and TIGIT (Fig. 6b). At the gene expression level, T_reg_ EM_2_ cells showed high expression of *IKZF3*, *CXCL13* and *ASCL2*, but low expression of *KLF2* and *IKZF2* (Fig. 6c). We used flow cytometry to confirm the presence of IKZF3 on protein level (Extended Data Fig. 7a) and the enrichment of IKZF3^+^ T_reg_ cells in MZL and FL using an independent cohort of 24 LN samples (Extended Data Fig. 7b). IKZF2, alias Helios, is well studied as a marker of natural T_reg_ cells^44^, but only few studies have explored the role of IKZF3, alias Aiolos, in T_reg_ cells. A previous study suggested that IKZF3^+^ T_reg_ cells usually lack IKZF2 and represent an inducible rather than natural T_reg_ cell subset with potent suppressive capacity^45^. On this basis, we intended to identify potential populations related to T_reg_ EM_2_ cells using scTCR data. We found that T_reg_ EM_2_ cells share a substantial proportion of clonotypes with T_FH_ cells (Fig. 6d), whereas this was not the case for other T_reg_ cell populations (Extended Data Fig. 7c). Based on these aspects, IKFZ3^+^ T_reg_ cells could resemble follicular regulatory T cells^46^.

We further investigated whether the proportion of T_reg_ EM_2_ cells was associated with clinical parameters. Lower-grade (1/2) FL had a higher proportion of T_reg_ EM_2_ cells (Fig. 6e, *P* = 0.002), whereas higher-grade FL (3A) had a higher proportion of T_FH_ cells (Fig. 6f, *P* = 0.03). We applied digital cytometry^39^ on the GALLIUM cohort^41,42^ to estimate the proportion of T_reg_ EM_2_ cells and T_FH_ cells, and to investigate whether these subsets were associated with progression-free survival. Indeed, there was a trend towards inferior prognosis in patients with higher proportions of T_FH_ cells (*P* = 0.05, Extended Data Fig. 7d), while no association was found for T_reg_ EM_2_ cells (*P* = 0.17, Extended Data Fig. 7e).

### CODEX captures the tumour and T cell microenvironment

**Extended Data Fig. 8:**
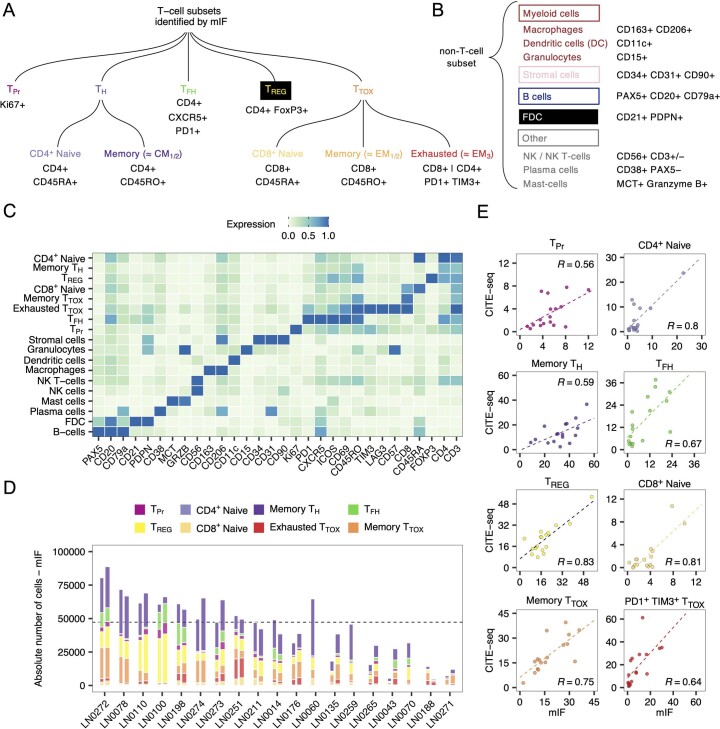
Profiles of cell types and T-cell subpopulations identified by multiplexed immunofluorescence. **a-b**) Overview of T-cell subsets (A) and other cell types (B) identified by highly multiplexed immunofluorescence including their marker profiles. **c**) Heatmap illustrating the mean expression of key marker proteins that were used to annotate the 18 cell types and T-cell subsets using highly multiplexed immunofluorescence. Expression values were scaled between 0 and 1. **d**) Shown are the absolute numbers of T-cells per subset, tissue core, and patient. A maximum number of two cores per patient were imaged. **e**) The low-granularity T-cell subpopulations detected by multiplexed immunofluorescence were aligned with the 14 high-granularity T-cell subsets identified by CITE-seq. Shown is the correlation of proportions for each of the T-cell subpopulations across n = 19 biologically indepdendent LN samples that were analyzed by both approaches. Pearson’s correlation coefficient is given for each panel (R). mIF: Multiplexed immunofluorescence. T_Pr_: Proliferating T-cells. T_H_: Helper T-cells. T_FH_: Follicular helper T-cells. T_REG_: Regulatory T-cells. T_TOX_: Cytotoxic T-cells. CM: Central memory. EM: Effector memory. FDC: Follicular dendritic cells. Source data

To localize T cell subsets in their spatial context, we used highly multiplexed immunofluorescence in formalin-fixed paraffin-embedded (FFPE) LN tissues using co-detection by indexing (CODEX)^47^. We established a panel of 50 antibodies (Supplementary Table 6) and imaged 35 FFPE biopsy cores from 19 patient samples (Extended Data Fig. 1a and Supplementary Table 1). Finally, we identified B cells, T cells, natural killer (NK) cells, NK T cells, mast cells, plasma cells, dendritic cells, follicular dendritic cells (FDCs) and stromal cells, as well as macrophages and granulocytes (Extended Data Fig. 8a–c). Among a total of around 5.5 million processed cells, we detected a median of approximately 45,000 T cells per tissue core (Extended Data Fig. 8d), which we further divided into eight subpopulations including naive CD4^+^ and CD8^+^ T cells (CD45RA^+^), T_FH_ cells (CD45RO^+^, PD1^+^, CXCR5^+^), T_reg_ cells (FOXP3^+^), memory CD4^+^ cells (CD45RO^+^), memory CD8^+^ T_tox_ cells (CD45RO^+^), exhausted T_tox_ cells (CD45RO^+^, PD1^+^, TIM3^+^) and T_pr_ cells (Ki67^+^, Extended Data Fig. 8a,c). A high-granularity classification of T cell subsets, as possible with the CITE-seq and flow cytometry, was hampered by lower signal-to-noise ratio (for example, CD62L and CD69) or reduced sensitivity of available antibodies (for example, IKZF3). Still, we aligned the low-granularity T cell subpopulations detected by multiplexed immunofluorescence with the 14 high-granularity T cell subsets identified by CITE-seq (Extended Data Fig. 8a), resulting in a median Pearson coefficient of 0.71 across all subpopulations (Extended Data Fig. 8e).

### B-NHL generates entity-specific microenvironmental patterns

**Fig. 7:**
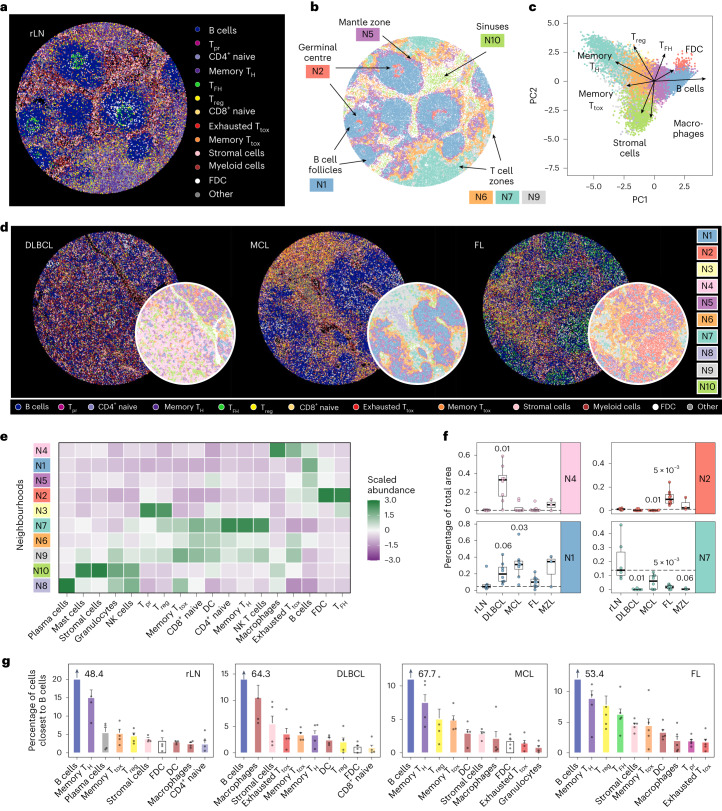
B-NHL disrupts the healthy LN architecture and generates entity-specific microenvironmental patterns. **a**,**b**, Each cell of a representative tumour-free LN-derived tissue core (rLN) is coloured by its subpopulation (**a**) or neighbourhood (**b**). **c**, Neighbourhoods shown in **b** were subjected to PCA. The top four loadings of components 1 and 2 are shown as vectors. **d**, Representative LN-derived tissue cores infiltrated by DLBCL, MCL or FL. Each cell is coloured by subpopulation or neighbourhood. **e**, Heatmap illustrating the mean and column-wise scaled abundance of subpopulations per neighbourhood across the complete mIF dataset. **f**, Box plots showing the proportions of selected neighbourhoods of each tissue core. Each entity and neighbourhood were tested versus rLN using the two-sided Wilcoxon test. *P* values were corrected for multiple testing using the Benjamini–Hochberg procedure. Only *P* values ≤0.05 are shown. Dashed lines indicate the median of tumour-free LNs (rLN). **g**, Bar plots showing percentages of cells that were located closest to B cells. DC, dendritic cells. Error bars represent s.e.m. For illustration purposes the B cell bar is not shown completely but indicated as number. In **e**–**g**, *N* = 19 biologically independent samples are shown. Source data

**Extended Data Fig. 9:**
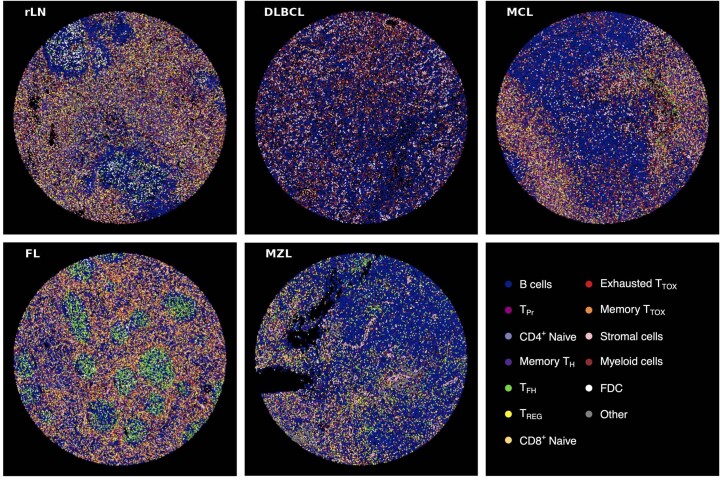
Representative lymph node tissue cores colored by T-cell subpopulation or non T-cell cell types. Shown are five representative LN tissue cores for all entities investigated. Each cell was colored by T-cell subsets or non T-cell cell types as indicated. FDC: Follicular dendritic cell. T_Pr_: Proliferating T-cells. T_H_: Helper T-cells. T_FH_: Follicular helper T-cells. T_REG_: Regulatory T-cells. T_TOX_: Cytotoxic T-cells. FDC: Follicular dendritic cells. Source data

In situ mapping of the above-mentioned cell types and T cell subpopulations enabled an intuitive visualization of tumour-free or malignant LN structure (Fig. 7a and Extended Data Fig. 9). To capture spatial organization systematically, we identified the 25 nearest neighbours of each cell by a sliding window approach and tabulated the frequencies of cell types and T cell subsets in each window. We used *k*-means clustering on the neighbour frequency tables to identify ten recurrent neighbourhoods (N1–N10; Fig. 7b) and PCA to identify the cell types most important for distinguishing N1 to N10 (Fig. 7c). Seven out of ten neighbourhoods captured important elements of intact LN architecture, including B cell follicles (N1) with T_FH_-rich and FDC-rich germinal centres (N2), follicle-surrounding mantle zones (N5), inter-follicular T cell zones (N6, N7 and N9) and sinuses (N10) harbouring predominantly stromal cells, macrophages, mast cells, granulocytes and NK cells (Fig. 7a–c), whereas N3, N4 and N8 could not be assigned to physiological LN regions.

**Extended Data Fig. 10:**
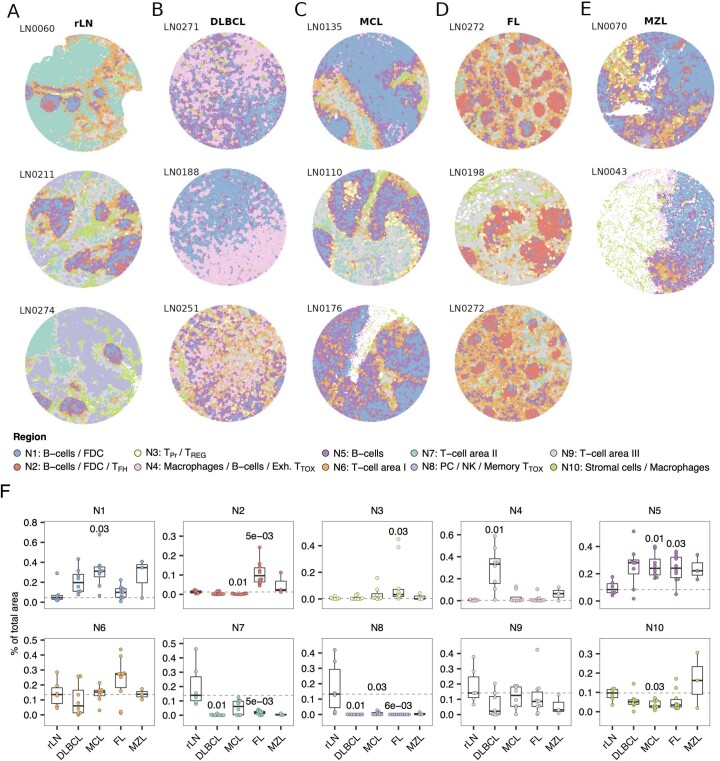
Qualitative and quantitative patterns of cellular neighbourhoods. **a–e**) Representative lymph node-derived tissue cores from three or two different patients per entity. Each cell was colored according to its neighbourhood. F) Box plots showing the proportions of selected neighborhoods of each tissue core across n = 19 biologically indepdent samples. Each entity and neighborhood was tested versus rLN using the two-sided Wilcoxon-test. P values were corrected for multiple testing using the Benjamini-Hochberg procedure. Only p values ≤ 0.05 are shown. Dashed lines indidcate the median of rLN. Box plots: centre line, median; box limits, first and third quartile; whiskers, 1.5 x interquartile range. rLN: Tumor-free lymph node. T_Pr_: Proliferating T-cells. T_FH_: Follicular helper T-cells. T_REG_: Regulatory T-cells. T_TOX_: Cytotoxic T-cells. FDC: Follicular dendritic cells. Source data

The described pattern was preserved in all tumour-free tissue cores (Extended Data Fig. 10a) but largely disrupted in B-NHL LNs (Fig. 7d and Extended Data Fig. 10b–e). LN-derived tissue cores infiltrated by DLBCL had the least degree of structure and exhibited a diffuse excess of a neighbourhood (N4) harbouring exhausted T cells, macrophages and tumour cells, whereas neighbourhoods (N7 and N8) rich in naive and memory T_H_ cells and T_tox_ cells were absent (Fig. 7d–f and Extended Data Fig. 10b). Tissue cores from LNs infiltrated by FL were characterized by expansion of germinal centre-like areas (N2) containing high numbers of (clonal) T_FH_ cells and FDC, surrounded by areas (N6 and N9) containing T_reg_, memory T_H_ cells, amd memory and exhausted T_tox_ cells, but also B cells (Fig. 7d–f and Extended Data Fig. 10d). In tissue cores infiltrated by MCL, we found a significant predominance of follicle-like B cell areas (N1, Fig. 7f), where T_FH_ cells and germinal centres were absent (Fig. 7d,e and Extended Data Fig. 10c). In contrast to DLBCL and FL, B cell areas in MCL were well separated and barely infiltrated by T cells (Fig. 7d and Extended Data Fig. 9), resulting in only little contact surface in the transition areas (N6, Fig. 7d,e). To identify interaction partners of B cells on the basis of spatial proximity, we determined the nearest neighbour of each B cell and ranked them by frequency (Fig. 7g). This analysis suggested strikingly different interaction partner across entities: While B cells derived from FL, MCL and tumour-free LNs were in closest contact to varying T cell subsets, for instance T_reg_ cells and T_FH_ cells in FL, we observed that B cells derived from DLBCL were mostly surrounded by macrophages and stromal and exhausted T cells (Fig. 7g).

## Discussion

Our study provides an in-depth and systematic reference map of LN-derived T cells in DLBCL, FL, MCL, MZL and tumour-free samples. Across all entities, malignant LNs were characterized by loss of CD4^+^ and CD8^+^ naive T cells, as well as CD69^−^ T_H_ CM_1_ cells and CD69^+^ T_H_ CM_2_ cells, but to variable extents harboured clonally expanded CD4^+^ and CD8^+^ PD1^+^ TIM3^+^ T_tox_ cells. The latter were part of a cellular trajectory that continuously converged into terminally exhausted T cells and had lost *TCF7* transcription factor activity^48^. Higher proportions of these cells were associated with inferior survival in FL and DLBCL, which is in line with previous studies in DLBCL^49^ or FL^50^. PD1^+^ TIM3^+^ T_tox_ cells were located within several spatial neighbourhoods, thereby allowing for contact with various types of immune cell. Specifically in LNs infiltrated by DLBCL, PD1^+^ TIM3^+^ T_tox_ cells were strongly co-localized with tumour cells and macrophages, which have recently been shown to be attracted by exhausted T cells and inversely, to reinforce T cell exhaustion^51^. This observation might explain why DLBCL harboured the highest proportions of PD1^+^ TIM3^+^ T_tox_ cells and why higher numbers of macrophages are associated with inferior outcome in DLBCL^52,53^.

A second trajectory was characterized by expression of PD1, absence of TIM3 and other inhibitory receptors, but maintained transcription factor activity of *TCF7*. Previous work suggested that only TCF7^+^ PD1^+^ T cells can be reinvigorated upon checkpoint inhibition, whereas terminally exhausted TCF7^−^ T cells cannot be restored^54,55^. This observation is of particular interest because immune checkpoint blockade is remarkably ineffective in nodal B-NHL^56,57^, which might be—in the light of our study—due to the predominance of terminally exhausted T cells. We speculate that patients with B-NHL harbouring high levels of PD1^+^ TIM3^−^ T_tox_ cells could represent a subgroup that benefits most from combining T cell-engaging immunotherapies and immune checkpoint blockade.

Beyond clonal T_tox_ cells, we found that both FL and MZL were characterized by clonal T_FH_ cells and IKZF3^+^ T_reg_ EM_2_ cells, and overall had a similar pattern of T cell infiltration. Whereas T_FH_ cells have been extensively studied and are known to support the growth of malignant B cells in FL^43^, an enrichment of IKZF3^+^ T_reg_ EM_2_ cells has, to our knowledge, neither been described in FL nor in MZL. We observed higher proportions of IKZF3^+^ T_reg_ EM_2_ cells in patients with low-graded FL, suggesting that this T cell subset could modulate the proliferation capacity of malignant B cells. IKZF3^+^ T_reg_ EM_2_ cells are suggested to bear strong suppressive capacity and represent an induced T_reg_ phenotype^45^. Indeed, we demonstrated that IKZF3^+^ T_reg_ EM_2_ cells and T_FH_ cells carry a substantial proportion of identical TCR clonotypes, which implies that IKZF3^+^ T_reg_ EM_2_ cells most likely derive from T_FH_ cells. More mechanistically oriented studied are needed to clarify the role of IKZF3^+^ T_reg_ cells.

In summary, our work refines previous knowledge of lymphoma-infiltrating T cells by employing recent technological advances and offers a different perspective on various B-NHL entities. This broader yet more detailed view revealed that B-NHL entities shape their T cell microenvironment in distinct manners, which could not be readily detected in studies investigating only single entities.

## Methods

### LN samples

Our study was approved by the Ethics Committee of the University of Heidelberg (S-254/2016). Informed consent of all patients was obtained in advance. As collection of LN samples was part of routine diagnostic procedures, participants were not compensated. LN patient samples were processed and frozen until further analysis^58^. In brief, whole LNs were cut into small pieces of approximately 1–2 mm size. LN cells were gently washed out by rinsing the pieces several times. After centrifugation, cells were frozen until further analysis. We did not include LN samples that showed obvious contamination of red blood cells after isolation. Also, samples from patients after allogeneic stem cell transplantation, chimaeric antigen receptor T cell or bispecific antibody therapy were not used in this study to minimize treatment-associated effects on the T cell microenvironment. For the same reason, samples were collected earliest 3 months after cessation of the last treatment.

### Single-cell 3′ RNA-seq and epitope expression profiling

Cells were thawed and immediately washed to remove dimethyl sulfoxide. To prevent entity-associated batch effects, samples were processed in batches of four to five containing at least three different entities. After thawing, we applied a dead cell removal kit (Miltenyi Biotec, 130-090-101) to all samples to reach a viability of at least 85–90%. Samples not reaching a viability above 85% were excluded. Then, 5 × 10^5^ viable cells were stained by a pre-mixed cocktail of oligonucleotide-conjugated antibodies (Supplementary Table 2) and incubated at 4 °C for 30 min. Cells were washed three times with ice-cold washing buffer and each time centrifuged at 4 °C for 5 min. After completion, cells were counted and viability was determined again. Samples not reaching a viability above 85% were excluded. The preparation of the bead–cell suspensions, synthesis of complementary DNA and single-cell gene expression and antibody-derived tag (ADT) libraries were performed using a Chromium single-cell v3.1 3′ kit (10x Genomics, 1000269) according to the manufacturer’s instructions.

### Single-cell 5′ RNA-seq and TCR repertoire profiling

Apart from epitope staining, sample processing was identical to 3′ scRNA-seq. The preparation of the bead-cell suspensions, synthesis of complementary DNA and single-cell gene expression and TCR libraries were performed using a Chromium single-cell v2 5′ and human TCR amplification kit (both 10x Genomics, 1000265, 1000252) according to the manufacturer’s instructions.

### Single-cell library sequencing and data processing

The 3′ gene expression and ADT libraries were pooled in a ratio of 3:1 aiming for 40,000 reads (gene expression) and 15,000 reads per cell (ADT), respectively. Sequencing was performed on a NextSeq 500 (Illumina). 5′ gene expression libraries were sequenced on a NextSeq 2000 (Illumina) aiming for 50,000 reads per cell. TCR libraries were sequenced on a NextSeq 500 (Illumina) aiming for a minimum of 5,000 reads per cell. After sequencing, the Cell Ranger (10x Genomics, v6.1.1) function cellranger mkfastq was used to demultiplex and to align raw base-call files to the reference genome (hg38). For 3′ gene and epitope expression libraries, the obtained FASTQ files were counted by the cellranger count command, whereas cellranger multi was used for 5′ gene expression and TCR libraries. As reference for TCR libraries, the VDJ ensembl reference (hg38, v5.0.0) was used. If not otherwise indicated, default settings were used for all functions.

### Analysis and integration of CITE-seq data

The R package Seurat (v4.1.0) was used to perform data quality control, filtering, and normalization. Gene counts per cell, ADT counts per cell and percentages of mitochondrial reads were computed using the built-in functions. PCA, shared nearest neighbour-based clustering and UMAP were done on the basis of the combined transcriptome and epitope data. After mapping the CD3 and CD19 epitope expression, non-T-cell clusters and doublets were removed. For data integration across the different preparation batches, we used the IntegrateData function of the Seurat package. For multimodal clustering based on gene and epitope expression, the weighted nearest neighbour approach was used^22^. To estimate the proportion of positive cells for each surface marker, we calculated the denoised protein expression using the totalVI Python package^59^.

### Inferring transcription factor activity on the basis of single-cell gene expression

We used the SCENIC (Single-Cell rEgulatory Network Inference and Clustering) package^23^ to infer gene regulatory networks and transcription factor activity on the basis of scRNA-seq data. Functions were used according to publicly available vignettes.

### Surface and intracellular flow cytometry staining

LN-derived cells were thawed and stained for viability using a fixable viability dye e506 (Thermo Fisher Scientific, 65-0866-14) and for different surface markers depending on the experimental set-up. The following surface antibodies were used: anti-CD3-PerCP-Cy5.5, anti-CD4-PE-Dazzle, anti-CD8-APC-Cy7, anti-CD45RA-FITC, anti-CD25-BV421, anti-CD31-BV605, anti-CXCR5-BV711, anti-TIM3-BV711, anti-CD278-BV605, anti-PD1-PE-Cy7, anti-CD69-AF700 and anti-CD244-BV421 (all BioLegend, Supplementary Table 4). For subsequent intracellular staining, cells were fixed and permeabilized with the intracellular fixation/permeabilization buffer set (Thermo Fisher Scientific, 88-8824-00) and stained with anti-Ki67-BV785, anti-FOXP3-AF647, anti-IKZF3-PE or adequate isotype controls (Thermo Fisher Scientific, BD Biosciences, Supplementary Table 4). Then, cells were analysed using an LSR Fortessa (BD Biosciences) and FACSDiva (BD Biosciences, version 8). For analysis and gating of flow cytometry data, FlowJo (v10.8.0) was used.

### Multinomial classification of T cell subpopulations

First, we evaluated whether multimodally defined T cell subsets can be distinguished in general by using surface markers only. Therefore, we trained gradient boosting models (‘xgbTree’)^28^ on the basis of surface marker expression of single-cell data. To reduce data load, only 30% of all cells were used. Tenfold cross-validation was employed to optimize the model. Since surface marker were not sufficient to reach sufficient accuracy, additional models were trained using surface marker plus gene expression of MKI67, IKZF3 and FOXP3, since these genes were differentially expressed between T cell subsets that could not be sufficiently predicted using only surface proteins. Finally, markers were ranked and selected by their variable importance to build flow cytometry panels. In case two or more markers deliver similar information (for example, CD95 and CD45RA), only one was selected. Gating strategies were built using the R package hypergate^29^ and optimized in an iterative process. The final gating strategy for all multimodally defined T cell subsets is illustrated in Supplementary Figs. 1 and 2.

### Prediction of B-NHL entity from T cell proportions

To assess feasibility of predicting B-NHL entity or tumour-free condition based on the proportions of all T cell subsets and overall T cell frequency, we trained classifiers based on multivariate regression with L1 penalty (LASSO) implemented in the R package glmnet (v4.1)^60^. The hyperparameter lambda was determined using cv.glmnet with balanced folds and weights inversely proportional to class size. The confusion matrix was computed using leave-one-out cross-validation.

### Analysis of TCR diversity

To estimate the diversity of the TCR repertoire on the basis of scTCR profiling, we employed the R package immunarch (v0.8.0; https://immunarch.com/) on the output files of the cellranger pipeline. TCR diversity across samples was compared using a rarefraction analysis. Therefore, the function repDiversity with method = ‘raref’ was applied.

### Mapping of 5′ scRNA-seq data onto CITE-seq reference data

To evaluate scTCR data in the context of multimodally defined T cell subsets, 5′ scRNA-seq data were mapped onto the CITE-seq reference data, using the built-in functions FindTransferAnchors and MapQuery of the Seurat package (v4.1.0). The mapping accuracy was evaluated by comparing the T cell subset proportion of 5′ and 3′ data of the identical patient sample.

### Pseudotime analysis and exhaustion signature

Pseudotime analysis based on gene expression profiles of T_tox_ cells was performed using the monocle3 package^32^. In brief, the Seurat object was converted into a cell dataset. Trajectory and pseudotime analysis were performed using the functions learn_graph and order_cells, respectively. As root cells, naive CD8^+^ T cells were selected. Minimal branch length was set to 10; otherwise, default settings were used.

To define a transcriptional module for T cell exhaustion, differentially expressed genes of terminally exhausted T cells, meaning T_tox_ cells with highest levels of inhibitory receptors and decreasing expression of effector molecules (Fig. 5c), were determined (Supplementary Table 5). The UCell package (v1.3.1)^61^, which is based on the Mann–Whitney *U* statistic, was then applied to calculate an exhaustion score for each cell.

### Deconvolution of bulk RNA sequencing data

Deconvolution of bulk RNA-seq data was performed using the interactive web application (https://cibersortx.stanford.edu/) developed by Newman and colleagues^39^. First, a signature matrix was created on the basis of the scRNA-seq data and the cluster annotation as cell types. Minimum expression was set to zero, and 200 replicates were used. Otherwise, default settings were applied to create a signature matrix. Second, cell fractions were imputed using bulk RNA-seq data and signature matrix as input. S-mode batch correction and absolute mode was used for each analysis.

### Survival analysis

Survival data were obtained only from previously published data or studies^2,4,41^. Analysis of the progression-free survival probability was performed in combination with the estimated cell type proportions using deconvolution of bulk RNA-seq data. To divide the data into two groups (low and high), we determined a cut-off based on the maximized *P* value of a log-rank test using the maxstat R package (v0.7.25). Kaplan–Meier curves were drawn using the survminer R package (v0.4.9).

### Tissue microarray and coverslip preparation

Representative tumour or tumour-free LN areas in archival FFPE tissue blocks from 19 patients (Extended Data Fig. 1a and Supplementary Table 1) were selected by board-certified pathologists of the Tissue Bank of the National Center for Tumor Diseases and Institute of Pathology at the University Hospital Heidelberg. Tissue microarrays (TMAs) containing two 4.5-mm cores per patient were generated. TMA sections (4 μm) were mounted onto Vectabond-precoated 25 × 25 mm coverslips and coated in paraffin for storage until staining^62^.

### Antibody conjugation, validation and titration

Multicolour immunofluorescence was performed using the CODEX approach^47^. Antibodies used for CODEX experiments are summarized in Supplementary Table 6. Purified, carrier-free antibodies (50–100 μg per reaction) were reduced with tris(2-carboxyethyl)phosphine and conjugated at 1:2 weight/weight ratio to maleimide-modified CODEX DNA oligonucleotides, which were purchased from TriLink Biotechnologies and deprotected via retro-Diels–Alder reaction. Conjugated antibodies were first evaluated in CODEX singleplex stains on tonsil and/or lymphoma tissue by comparison with online databases (The Human Protein Atlas, Pathology Outlines), immunohistochemical reference stains and/or published literature under the supervision of a board-certified pathologist. Staining patterns were validated in multiplex experiments in the presence of positive and negative control antibodies and the appropriate dilution of each antibody was titrated starting from 1:100 to optimize signal-to-noise ratio.

### Multiplex tissue staining and fixation

Coverslips were deparaffinized, rehydrated and submitted to heat-induced epitope retrieval at pH 9 (Dako target retrieval solution, S236784-2, Agilent) and 97 °C for 10 min in a Lab Vision PT module (Thermo Fisher). After blocking of non-specific binding with CODEX FFPE blocking solution, coverslips were stained overnight with the full antibody panel at the dilutions given in Supplementary Table 6 in CODEX FFPE blocking solution^63^ in a sealed humidity chamber at 4 °C on a shaker. Coverslips were then fixed with 1.6% paraformaldehyde, followed by methanol and BS3 fixative (Thermo Fisher) before storage in CODEX buffer S4 until imaging^63^.

### Multicycle imaging

Stained coverslips were mounted onto custom acrylic plates (Pololou Corporation) with mounting gaskets (Qintay), thereby creating a flow cell with a surface area of 19 × 19 mm above the tissue for fluid exchange. Acrylic plates were inserted into a Keyence BZ-X710 inverted fluorescence microscope equipped with a CFI Plan Apo λ 20×/0.75 objective (Nikon) using custom adapters. For each core an area of 7 × 7 fields of view (30% overlap between tiles) and an adequate number of *z* planes (10–14) required to capture the best focal plane across the imaging area were selected. Multicycle imaging was performed using a CODEX microfluidics device and CODEX driver software v1.29.0.1 (Akoya Biosciences). Exposure times and assignment of markers to imaging cycles and fluorescent channels are provided in Supplementary Table 6. After completion of multicycle imaging, coverslips were stained with haematoxylin–eosin, and the same areas were imaged in brightfield mode.

### Image processing

Raw TIFF images were processed using the RAPID pipeline^64^ in MATLAB (version R2020a) with the following settings: nCyc = 51, nReg = number of regions imaged (depending on TMA), nTil = 49, nZ = number of *z*-planes imaged (depending on TMA), nCh = [1,4], nTilRow = 7, nTilCol = 7, overlapRatio = 0.3, reg_range = 1:nReg, cyc_range = 1:nCyc, til_range = 1:nTil, cpu_num = depending on computer system used, neg_flag = 1, gpu_id = depending on number of GPUs available, cyc_bg = 1. After deconvolution (two iterations), best focal plane selection, lateral drift compensation, stitching of individual images and background subtraction, processed images were concatenated to hyperstacks. All tissue cores were checked visually for staining quality of each antibody in ImageJ/Fiji (version 1.53q).

### Cell segmentation and cell type annotation

Individual nuclei were segmented on the basis of the Hoechst stain (cycle 1), derived nuclear masks were dilated, and cellular marker expression levels were quantified using a modified version of the Mask region-convolutional neural network-based CellSeg software^65^ run on the full-resolution RAPID stitched images with the following parameters: GROWTH_PIXELS_PLANE = 1.0, output_adjacency_quant = True, BOOST = 1, OVERLAP = 80, MIN_AREA = 80, INCREASE_FACTOR = 4.0, AUTOBOOST_PERCENTILE = 99.98. A threshold based on the intensity of the nuclear markers Hoechst and DRAQ5 was used to exclude non-cellular events and remove cells from tissue areas of low image quality. Marker expression levels compensated for lateral spillover were used for further analysis and the range of each marker was *z* normalized per imaging run. A total of *n* = 5,690,284 cells were submitted to an initial round of Leiden-based clustering (n_neighbors = 10, resolution = 2) on key phenotypic markers (CD11b, CD11c, CD14, CD15, CD16, CD163, CD20, CD206, CD21, CD25, CD3, CD31, CD34, CD38, CD4, CD45, CD5, CD56, CD57, CD68, CD7, CD79a, CD8, CD90, FOXP3, HLA-DR, kappa light chain, lambda light chain, MCT, PAX5 and PDPN) using the scanpy Python package^66,67^. Each of the resulting clusters (*n* = 55) was assessed for purity of its cell type composition on the basis of marker expression and overlays of the cells in each cluster onto image hyperstacks using CODEX scripts for ImageJ/Fiji (available at ref. ^68^). Clusters were merged, split and/or further subclustered as appropriate to define broad cell types (for example, T cells, B cells and so on). This process was repeated within these cell types using additional markers as appropriate to annotate more granular cell subsets. Briefly, marker combinations used for cell typing of non-T cells include CD16, CD68, CD163, CD206 and HLA-DR (macrophages); CD11c, CD68 and HLA-DR (DC); CD15 (granulocytes); MCT and GRZB (mast cells); CD34, CD31, CD90 and PDPN (stromal cells); PDPN and CD21 (FDC); CD56 (NK and NK T cells); CD38, CD31, and kappa and lambda light chain (plasma cells); PAX5, CD20 and CD79a (B cells). Further, T cell subsets were derived on the basis of key subset markers (CD45, CD45RA, CD45RO, CD3, CD5, CD7, CD4, CD8, FOXP3, CXCR5, CXCL13, PD1, TIM3, CD31 and Ki67) and were validated using expression of additional T cell markers in the panel and overlays onto image hyperstacks.

### Neighbourhood and nearest neighbour analysis

Neighbourhood analysis was modified based on a previously described approach^47^. For each cell of the joint highly multiplexed immunofluorescence data, the 25 nearest neighbours were determined on the basis of their Euclidean distance of the *x* and *y* coordinates, resulting in one ‘window’ of cells per individual cell. Next, these windows were grouped using *k*-means clustering based on the cell type proportions within each window. Finally, each cell was annotated by the neighbourhood of its surrounding ‘window’. *k* = 10 was selected on the basis of the overlays of the neighbourhood assignments with the original fluorescent and haematoxylin–eosin-stained images. Higher values of *k* did not result in an improved biologically interpretable number of neighbourhoods.

### Interactive browsing of highly multiplexed immunofluorescence data

All tissue cores imaged in this study including staining of 52 different markers are available for interactive browsing at http://45.88.80.143:8501/.

### Statistics and reproducibility

Statistical analysis and data illustration was performed in R (v4.2.1) using the R packages glmnet (v4.1-2), ggplotify (v0.1.0), maxstat (v0.7-25), R.oo (v1.24.0), rstatix (v0.7.0), viridis (v0.6.2), dplyr (v1.0.10), tidyverse (v1.3.1), FNN (v1.1.3), Matrix (v1.5-1), ggraph (v2.0.6), survival (v3.2-13), R.methodsS3 (v1.8.1), ggpubr (v0.4.0), viridisLite (v0.4.1), purrr (v0.3.4), future.apply (v1.8.1), immunarch (v0.7.0), igraph (v1.3.5), survminer (v0.4.9), readxl (v1.4.1), ggrepel (v0.9.1), SeuratObject (v4.0.4), readr (v2.1.2), future (v1.23.0), data.table (v1.14.2), ggrastr (v1.0.1), ggridges (v0.5.3), caret (v6.0-90), matrixStats (v0.61.0), Seurat (v4.1.0), tidyr (v1.2.1), pamr (v1.56.1), dtplyr (v1.2.2), ggtext (v0.1.1), cowplot (v1.1.1), lattice (v0.20-45), scales (v1.2.1), forcats (v0.5.1), tibble (v3.1.8), cluster (v2.1.2), rmdformats (v1.0.4), ggalluvial (v0.12.3), R.utils (v2.11.0), patchwork (v1.1.2), RColorBrewer (v1.1-3), stringr (v1.4.1) and ggplot2 (v3.3.6). The computational codes to exactly reproduce all analysis steps and figures is provided below (see ‘Code availability statement’). No statistical method was used to determine sample size before data collection. No data were excluded from the analyses. The experiments were not randomized. The investigators were not blinded to allocation during experiments and outcome assessment.

### Reporting summary

Further information on research design is available in the Nature Portfolio Reporting Summary linked to this article.

## Online content

Any methods, additional references, Nature Portfolio reporting summaries, source data, extended data, supplementary information, acknowledgements, peer review information; details of author contributions and competing interests; and statements of data and code availability are available at 10.1038/s41556-024-01358-2.

### Supplementary information

Supplementary Information Supplementary Figs. 1 and 2.

Reporting Summary

Supplementary Table 1 Supplementary Tables 1–6.

### Source data

Source Data Fig. 1 Statistical source data.

Source Data Fig. 2 Statistical source data.

Source Data Fig. 3 Statistical source data.

Source Data Fig. 4 Statistical source data.

Source Data Fig. 5 Statistical source data.

Source Data Fig. 6 Statistical source data.

Source Data Fig. 7 Statistical source data.

Source Data Extended Data Fig. 1 Statistical source data.

Source Data Extended Data Fig. 2 Statistical source data.

Source Data Extended Data Fig. 3 Statistical source data.

Source Data Extended Data Fig. 4 Statistical source data.

Source Data Extended Data Fig. 5 Statistical source data.

Source Data Extended Data Fig. 6 Statistical source data.

Source Data Extended Data Fig. 7 Statistical source data.

Source Data Extended Data Fig. 8 Statistical source data.

Source Data Extended Data Fig. 9 Statistical source data.

Source Data Extended Data Fig. 10 Statistical source data.

## Data Availability

RNA-seq, epitope and TCR data that support the findings of this study have been deposited in the Gene Expression Omnibus (GEO) under accession codes GSE252608 and GSE252455. Highly multiplexed immunofluorescence images will be available in the BioStudies database (https://www.ebi.ac.uk/biostudies/) under accession number S-BIAD565 (ref. ^69^). Flow cytometry data have been deposited in figshare under 10.6084/m9.figshare.24915633. Source data are provided with this paper.

## Code availability

The computational codes, in the form of Rmarkdown documents, for reproducing all figures are available on GitHub (https://github.com/Huber-group-EMBL/CITEseqLN-Tcells).
