# Supplementary material for: Multimodal and spatially resolved profiling identifies distinct patterns of T cell infiltration in nodal B cell lymphoma entities
Source: Nat Cell Biol. 2024 Feb 20;26(3):478–89. doi: 10.1038/s41556-024-01358-2 (PMC10940160; doi:10.1038/s41556-024-01358-2)
Supplement: Supplementary file 1 — Supplementary Figs. 1 and 2. [file 41556_2024_1358_MOESM1_ESM.pdf]

nature cell biology

Resource https://doi.org/10.1038/s41556-024-01358-2

Multimodal and spatially resolved profiling identifies distinct patterns of T cell infiltration in nodal B cell lymphoma entities

In the format provided by the authors and unedited

Supplementary Figure 1

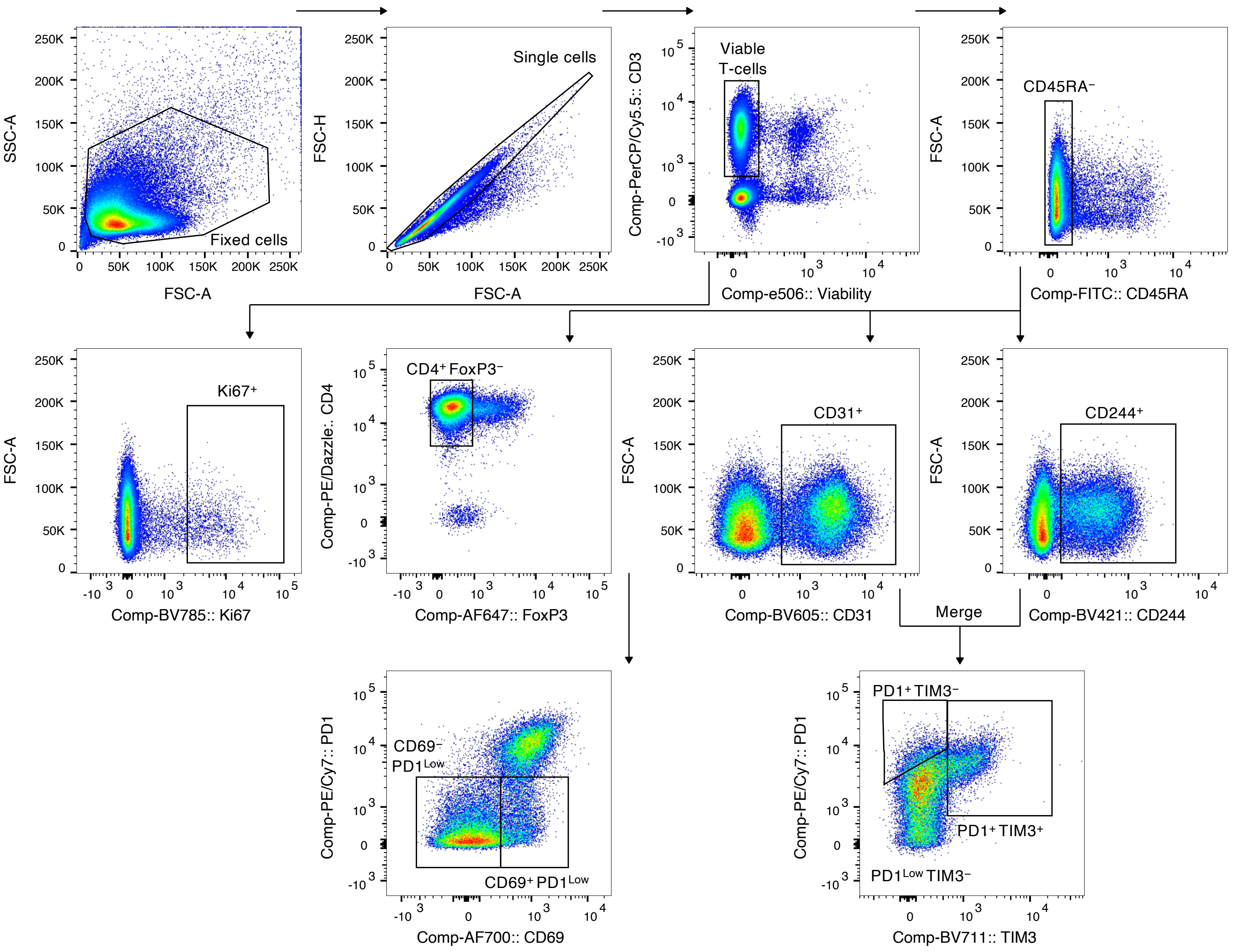

Supplementary Figure 1.

Flow cytometry gating strategy for one representative lymph node sample (see method section for details) for T_{Pr} (Ki67$^+$), T_H CM_1 (CD69$^-$ PD1^{Low}), T_H CM_2 (CD69$^+$ PD1^{Low}), T_{TOX} EM_1 (PD1^{Low} TIM3$^-$), T_{TOX} EM_2 (PD1$^+$ TIM3$^-$), and T_{TOX} EM_3 (PD1$^+$ TIM3$^+$) cells. T_{Pr}: Proliferating T-cells. T_H: Helper T-cells. T_{REG}: Regulatory T-cells. T_{TOX}: Cytotoxic T-cells. T_{DN}: Double negative T-cells. CM: Central memory. EM: Effector memory.

Supplementary Figure 2

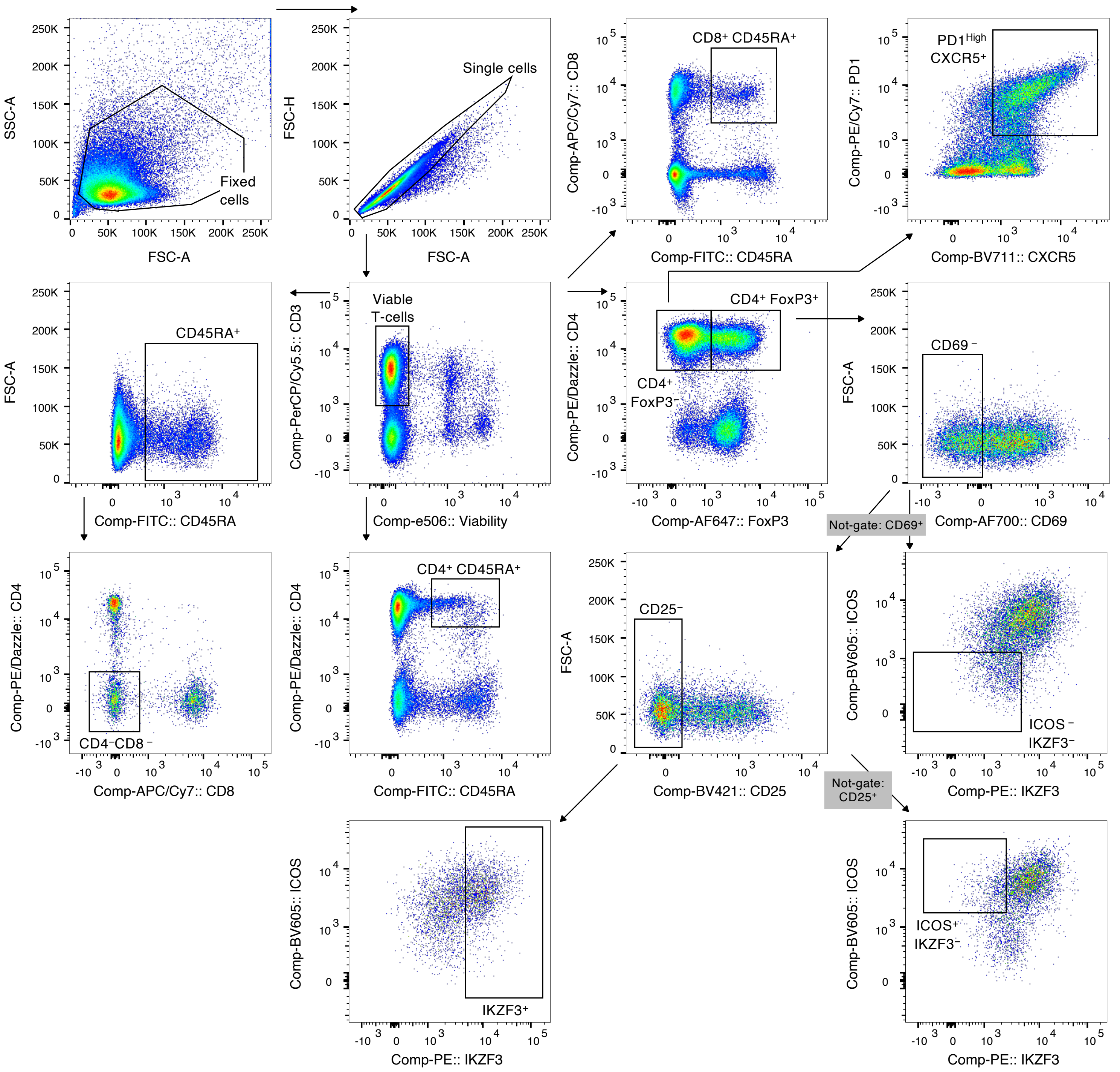

Supplementary Figure 2.

Flow cytometry gating strategy (see method section for details) for one representative lymph node sample for T_H Naïve ($CD4^+$ $CD45RA^+$), T_{TOX} Naïve ($CD8^+$ $CD45RA^+$), T_{DN} ($CD4^-$ $CD8^-$), T_{FH} ($PD1^{High}$ $CXCR5^+$), T_{REG} CM_1 ($CD69^-$), T_{REG} CM_2 ($ICOS^+$ $IKZF3^-$), T_{REG} EM_1 ($ICOS^-$ $IKZF3^-$), and T_{REG} EM_2 ($IKZF3^+$). T_{Pr}: Proliferating T-cells. T_{FH}: Follicular helper T-cells. T_{REG}: Regulatory T-cells. T_{DN}: Double negative T-cells. CM: Central memory. EM: Effector memory.
