# Supplementary material for: Multimodal and spatially resolved profiling identifies distinct patterns of T cell infiltration in nodal B cell lymphoma entities
Source: Nat Cell Biol. 2024 Feb 20;26(3):478–89. doi: 10.1038/s41556-024-01358-2 (PMC10940160; doi:10.1038/s41556-024-01358-2)
Supplement: Supplementary file 2 — Reporting Summary [file 41556_2024_1358_MOESM2_ESM.pdf]

nature portfolio

Corresponding author(s): Wolfgang Huber, Sascha Dietrich

Last updated by author(s): Jan 10, 2024

Reporting Summary

Nature Portfolio wishes to improve the reproducibility of the work that we publish. This form provides structure for consistency and transparency in reporting. For further information on Nature Portfolio policies, see our Editorial Policies and the Editorial Policy Checklist.

Statistics

For all statistical analyses, confirm that the following items are present in the figure legend, table legend, main text, or Methods section.

n/a	Confirmed	
☐	☒	The exact sample size (*n*) for each experimental group/condition, given as a discrete number and unit of measurement
☐	☒	A statement on whether measurements were taken from distinct samples or whether the same sample was measured repeatedly
☐	☒	The statistical test(s) used AND whether they are one- or two-sided<br>*Only common tests should be described solely by name; describe more complex techniques in the Methods section.*
☒	☐	A description of all covariates tested
☐	☒	A description of any assumptions or corrections, such as tests of normality and adjustment for multiple comparisons
☐	☒	A full description of the statistical parameters including central tendency (e.g. means) or other basic estimates (e.g. regression coefficient) AND variation (e.g. standard deviation) or associated estimates of uncertainty (e.g. confidence intervals)
☐	☒	For null hypothesis testing, the test statistic (e.g. *F*, *t*, *r*) with confidence intervals, effect sizes, degrees of freedom and *P* value noted<br>*Give P values as exact values whenever suitable.*
☒	☐	For Bayesian analysis, information on the choice of priors and Markov chain Monte Carlo settings
☒	☐	For hierarchical and complex designs, identification of the appropriate level for tests and full reporting of outcomes
☐	☒	Estimates of effect sizes (e.g. Cohen's *d*, Pearson's *r*), indicating how they were calculated

Our web collection on statistics for biologists contains articles on many of the points above.

Software and code

Policy information about availability of computer code

Data collection	Flow cytometry data were collected using FACSDiva (version 8, BD Biosciences).<br>Multiplexed immunofluorescence data were recorded using CODEX driver software (version 1.29.0.1, Akoya Biosciences).<br>Raw TIFF images were processed using the RAPID pipeline in Matlab (version R2020a).<br>Single cell data were processed using CellRanger pipelines (10x Genomics, version 6.1.1).
Data analysis	FlowJo (v10.8.0).<br>ImageJ/Fiji (v1.53q).<br>Scanpy (v1.8.2)<br>Cellseg (v2022)<br>R (v4.2.1)<br>The computational codes, in the form of Rmarkdown documents, for reproducing all main and supplementary figures is available on GitHub (github.com/Huber-group-EMBL/CITEseqLN-Tcells.git). This contains also all R packages including version numbers used for data analysis.<br>R packages<br>knitr (v1.40)<br>glmnet (v4.1-2)<br>ggplotify (v0.1.0)<br>maxstat (v0.7-25)<br>R.oo (v1.24.0)<br>rstatix (v0.7.0)<br>viridis (v0.6.2)<br>dplyr (v1.0.10)

tidyverse (v1.3.1)
FNN (v1.1.3)
Matrix (v1.5-1)
ggraph (v2.0.6)
survival (v3.2-13)
R.methodsS3 (v1.8.1)
ggpubr (v0.4.0)
viridisLite (v0.4.1)
purrr (v0.3.4)
future.apply (v1.8.1)
immunarch (v0.7.0)
igraph (v1.3.5)
survminer (v0.4.9)
readxl (v1.4.1)
ggrepel (v0.9.1)
SeuratObject (v4.0.4)
readr (v2.1.2)
future (v1.23.0)
data.table (v1.14.2)
ggrastr (v1.0.1)
ggridges (v0.5.3)
caret (v6.0-90)
matrixStats (v0.61.0)
Seurat (v4.1.0)
tidyr (v1.2.1)
pamr (v1.56.1)
dtplyr (v1.2.2)
ggtext (v0.1.1)
cowplot (v1.1.1)
lattice (v0.20-45)
scales (v1.2.1)
forcats (v0.5.1)
tibble (v3.1.8)
cluster (v2.1.2)
rmdformats (v1.0.4)
ggalluvial (v0.12.3)
R.utils (v2.11.0)
patchwork (v1.1.2)
RColorBrewer (v1.1-3)
stringr (v1.4.1)
ggplot2 (v3.3.6)

For manuscripts utilizing custom algorithms or software that are central to the research but not yet described in published literature, software must be made available to editors and reviewers. We strongly encourage code deposition in a community repository (e.g. GitHub). See the Nature Portfolio guidelines for submitting code & software for further information.

Data

Policy information about availability of data

All manuscripts must include a data availability statement. This statement should provide the following information, where applicable:

- Accession codes, unique identifiers, or web links for publicly available datasets
- A description of any restrictions on data availability
- For clinical datasets or third party data, please ensure that the statement adheres to our policy

RNA–seq, epitope and TCR data that support the findings of this study have been deposited in the Gene Expression Omnibus (GEO) under accession codes GSE252608 and GSE252455. Highly multiplexed immunofluorescence images will be available in the BioStudies database (https://www.ebi.ac.uk/biostudies/) under accession number S-BIAD565 [88]. Flow cytometry data have been deposited in figshare under 10.6084/m9.figshare.24915633.

Human research participants

Policy information about studies involving human research participants and Sex and Gender in Research.

Reporting on sex and gender	None of our findings was specific to only one sex. A total number of 55 male and 46 female lymph node samples were investigated in this study. Sex was collected from patient charts and irrelevant for sample collection. Informed consent to collect sex as meta data was given in advance. Sex was investigated as covariate for T-cell subset abundance. No significant association with sex were detected.
Population characteristics	The patient characteristics which are relevant for this study are listed in detail in Supplementary Table 1.
Recruitment	Patient samples were selected based on the diagnosis (diffuse large B cell lymphoma, follicular lymphoma, marginal zone lymphoma, mantle cell lymphoma, reactive lymphadenitis) and the availability of high quality viable cells in our lymph node biobank. There is no self-selection bias or other biases in recruitment.

Ethics oversight | Ethics Committee of the University of Heidelberg

Note that full information on the approval of the study protocol must also be provided in the manuscript.

Field-specific reporting

Please select the one below that is the best fit for your research. If you are not sure, read the appropriate sections before making your selection.

☒ Life sciences ☐ Behavioural & social sciences ☐ Ecological, evolutionary & environmental sciences

For a reference copy of the document with all sections, see nature.com/documents/nr-reporting-summary-flat.pdf

Life sciences study design

All studies must disclose on these points even when the disclosure is negative.

Sample size: No statistical method was used to determine sample size prior to data collection. To estimate sufficient sample size, we performed an extensive literature research. We aimed for a sample size that exceeds 9 out of 10 studies using similar technical approaches on human samples as we used. The result was a sample size of around 10 samples per group / entity, i.e. 50 samples in total. Our core analysis about the quantitative infiltration patterns is even based on 100 samples. Further, in-depth experiments were performed on a subset of samples in order to confirm null hypothesis.

Data exclusions: No data were excluded from the analysis.

Replication: All experiments were independently replicated to verify reproducibility. The number of biological replicates is given for each figure.

Randomization: Not relevant. We do not have any experimental groups in our study.

Blinding: Not relevant. We do not have any experimental groups in our study.

Reporting for specific materials, systems and methods

We require information from authors about some types of materials, experimental systems and methods used in many studies. Here, indicate whether each material, system or method listed is relevant to your study. If you are not sure if a list item applies to your research, read the appropriate section before selecting a response.

Materials & experimental systems

n/a	Involved in the study	
☐	☒	Antibodies
☒	☐	Eukaryotic cell lines
☒	☐	Palaeontology and archaeology
☒	☐	Animals and other organisms
☒	☐	Clinical data
☒	☐	Dual use research of concern

Methods

n/a	Involved in the study	
☒	☐	ChIP-seq
☐	☒	Flow cytometry
☒	☐	MRI-based neuroimaging

Antibodies

Antibodies used:

CITE-seq
Target,Gene,Clone,Isotype,Supplier,Catalogue,BarcodeNo,Barcode,Dilution
CD10,MME,HI10a,Mouse IgG1 κ,Biolegend,312231,0062,CAGCCATTCATTAGG,1:4
CD103,ITGAE,Ber-ACT8 ,Mouse IgG1 κ,Biolegend,350231,0145,GACCTCATTGTGAAT,1:32
CD11b,ITGAM,ICRF44,Mouse IgG1 κ,Biolegend,301353,0161,GACAAGTGATCTGCA,1:10
CD11c,ITGAX,S-HCL-3,Mouse IgG2b κ,Biolegend,371519,0053,TACGCCTATAACTTG,1:16
CD127,IL7R,A019D5,Mouse IgG1 κ,Biolegend,351352,0390,GTGTGTTGTCCTATG,1:8
CD134,TNFRSF4,Ber-ACT35,Mouse IgG1 κ,Biolegend,350033,0158,AACCCACCGTTGTTA,1:4
CD137,TNFRSF9,4B4-1,Mouse IgG1 κ,Biolegend,309835,0355,CAGTAAGTTCGGGAC,1:4
CD150,SLAMF1,A12 (7D4),Mouse IgG1 κ,Biolegend,306313,0870,GTCATTGTATGTCTG,1:4
CD152,CTLA4,BNI3,Mouse IgG2a κ,Biolegend,369619,0151,ATGGTTCACGTAATC,1:4
CD16,FCGR3A,3G8,Mouse IgG1 κ,Biolegend,302061,0083,AAGTTCACTCTTTGC,1:8
CD161,KLRB1,HP-3G10,Mouse IgG1 κ,Biolegend,339945,0149,GTACGCAGTCCTTCT,1:4
CD183,CXCR3,G025H7,Mouse IgG1 κ,Biolegend,353745,0140,GCGATGGTAGATTAT,1:8
CD184,CXCR4,12G5,Mouse IgG2a κ,Biolegend,306531,0366,TCAGGTCCTTTCAAC,1:2
CD185,CXCR5,J252D4,Mouse IgG1 κ,Biolegend,356937,0144,AATTCAACCGTCGCC,1:4
CD19,CD19,HIB19,Mouse IgG1 κ,Biolegend,302259,0050,CTGGGCAATTACTCG,1:16

CD194,CCR4,L291H4,Mouse IgG1 κ,Biolegend,359423,0071,AGCTTACCTGCACGA,1:16
CD195,CCR5,J418F1,Rat IgG2b κ,Biolegend,359135,0141,CCAAAGTAAGAGCCA,1:4
CD197,CCR7,G043H7,Mouse IgG2a κ,Biolegend,353247,0148,AGTTCAGTCAACCGA,1:1.25
CD2,CD2,TS1/8,Mouse IgG1 κ,Biolegend,309229,0367,TACGATTTGTCAGGG,1:16
CD20,MS4A1,2H7,Mouse IgG2b κ,Biolegend,302359,0100,TTCTGGGTCCCTAGA,1:8
CD21,CR2,Bu32,Mouse IgG1 κ,Biolegend,354915,0181,AACCTAGTAGTTCGG,1:16
CD22,CD22,S-HCL-1,Mouse IgG2b κ,Biolegend,363514,0393,GGGTTGTTGTCTTTG,1:16
CD223,LAG3,11C3C65,Mouse IgG1 κ,Biolegend,369333,0152,CATTTGTCTGCCGGT,1:4
CD23,FCER2,EBVCS-5,Mouse IgG1 κ,Biolegend,338523,0897,TCTGTATAACCGTCT,1:4
CD24,CD24,ML5,Mouse IgG2a κ,Biolegend,311137,0180,AGATTCCTTCGTGTT,1:4
CD244,CD244,C1.7,Mouse IgG1 κ,Biolegend,329527,0189,TCGCTTGGATGGTAG,1:16
CD25,IL2RA,BC96,Mouse IgG1 κ,Biolegend,302643,0085,TTTGTCCTGTACGCC,1:8
CD27,CD27,O323,Mouse IgG1 κ,Biolegend,302847,0154,GCACTCCTGCATGTA,1:8
CD273,PDCD1LG2,24F.10C12,Mouse IgG2a κ,Biolegend,329619,0008,TCAACGCTTGGCTAG,1:8
CD274,PDCD1LG1,29E.2A3,Mouse IgG2b κ,Biolegend,329743,0007,GTTGTCCGACAATAC,1:4
CD278,ICOS,C398.4A,Armenian Hamster IgG,Biolegend,313555,0171,CGCGCACCCATTAAA,1:4
CD279,PDCD1,EH12.2H7,Mouse IgG1 κ,Biolegend,329955,0088,ACAGCGCCGTATTTA,1:8
CD28,CD28,CD28.2,Mouse IgG1 κ,Biolegend,302955,0386,TGAGAACGACCCTAA,1:4
CD29,ITGB1,TS2/16,Mouse IgG1 κ,Biolegend,303027,0369,GTATTCCCTCAGTCA,1:8
CD3,CD3E,UCHT1,Mouse IgG1 κ,Biolegend,300475,0034,CTCATTGTAACTCCT,1:8
CD31,PECAM1,WM59,Mouse IgG1 κ,Biolegend,303137,0124,ACCTTTATGCCACGG,1:8
CD32,FCGR2A,FUN-2,Mouse IgG2b κ,Biolegend,303223,0142,GCTTCCGAATTACCG,1:16
CD357,TNFRSF18,108-17,Mouse IgG2a κ,Biolegend,371225,0360,ACCTTTCGACACTCG,1:4
CD366,HAVCR2,F38-2E2,Mouse IgG1 κ,Biolegend,345047,0169,TGTCCTACCCAACTT,1:4
CD38,CD38,HIT2,Mouse IgG1 κ,Biolegend,303541,0389,TGTACCCGCTTGTGA,1:8
CD39,ENTPD1,A1,Mouse IgG1 κ,Biolegend,328233,0176,TTACCTGGTATCCGT,1:16
CD4,CD4,RPA-T4,Mouse IgG1 κ,Biolegend,300563,0072,TGTTCCCGCTCAACT,1:8
CD43,SPN,CD43-10G7,Mouse IgG1 κ,Biolegend,343209,0357,GATTAACCAGCTCAT,1:4
CD44,CD44,IM7,Rat IgG2b κ,Biolegend,103045,0073,TGGCTTCAGGTCCTA,1:16
CD45,PTPRC,HI30,Mouse IgG1 κ,Biolegend,304064,0391,TGCAATTACCCGGAT,1:40
CD45RA,PTPRC,HI100,Mouse IgG2b κ,Biolegend,304157,0063,TCAATCCTTCCGCTT,1:40
CD45RO,PTPRC,UCHL1,Mouse IgG2a κ,Biolegend,304255,0087,CTCCGAATCATGTTG,1:10
CD47,CD47,CC2C6,Mouse IgG1 κ,Biolegend,323129,0026,GCATTCTGTCACCTA,1:10
CD48,CD48,BJ40,Mouse IgG1 κ,Biolegend,336709,0029,CTACGACGTAGAAGA,1:8
CD5,CD5,UCHT2,Mouse IgG1 κ,Biolegend,300635,0138,CATTAACGGGATGCC,1:16
CD56,NCAM1,QA17A16,Mouse IgG1 κ,Biolegend,392421,0084,TTCGCCGCATTGAGT,1:10
CD57,B3GAT1,QA17A04,Mouse IgG1 κ,Biolegend,393319,0168,AACTCCCTATGGAGG,1:4
CD62L,SELL,DREG-56,Mouse IgG1 κ,Biolegend,304847,0147,GTCCCTGCAACTTGA,1:16
CD69,CD69,FN50,Mouse IgG1 κ,Biolegend,310947,0146,GTCTCTTGGCTTAAA,1:8
CD7,CD7,CD7-6B7,Mouse IgG2a κ,Biolegend,343123,0066,TGGATTCCCGGACTT,1:16
CD70,CD70,113-16,Mouse IgG1 κ,Biolegend,355117,0027,CGCGAACATAAGAAG,1:4
CD73,NT5E,AD2,Mouse IgG1 κ,Biolegend,344029,0577,CAGTTCCTCAGTTCG,1:4
CD79b,CD79B,CB3-1,Mouse IgG1 κ,Biolegend,341415,0187,ATTCTTCAACCGAAG,1:8
CD86,CD86,IT2.2,Mouse IgG2b κ,Biolegend,305443,0006,GTCTTTGTCAGTGCA,1:16
CD8a,CD8A,RPA-T8,Mouse IgG1 κ,Biolegend,301067,0080,GCTGCGCTTTCCATT,1:8
CD95,FAS,DX2,Mouse IgG1 κ,Biolegend,305649,0156,CCAGCTCATTAGAGC,1:8
Isotype Ctrl,,MOPC-21,Mouse IgG1 κ,Biolegend,400199,0090,GCCGGACGACATTAA,1:4
Isotype Ctrl,,HTK888,Armenian Hamster IgG,Biolegend,400973,0241,CCTGTCATTAAGACT,1:8
Isotype Ctrl,,MPC-11,Mouse IgG2b κ,Biolegend,400373,0092,ATATGTATCACGCGA,1:4
Isotype Ctrl,,RTK4530,Rat IgG2b κ,Biolegend,400673,0095,GATTCTTGACGACCT,1:4
Isotype Ctrl,,MOPC-173,Mouse IgG2a κ,Biolegend,400285,0091,CTCCTACCTAAACTG,1:8
Kappa,IGKC,MHK-49 ,Mouse IgG1 κ,Biolegend,316531,0894,AGCTCAGCCAGTATG,1:10
KLRG1,KLRG1,SA231A2,Mouse IgG2a κ,Biolegend,367721,0153,CTTATTTCCTGCCCT,1:8
Lambda,IGLC2,MHL-38 ,Mouse IgG2a κ,Biolegend,316627,0898,CAGCCAGTAAGTCAC,1:10
TIGIT,TIGIT,A15153G,Mouse IgG2a κ,Biolegend,372725,0089,TTGCTTACCGCCAGA,1:16

Flow Cytometry
Target,Conjugate,Alternative,Clone,Isotype,Supplier,Catalogue,Dilution
CD25,BV421,,BC96,Mouse IgG1 κ,Biolegend,302629,1:25
CD278,BV605,ICOS,WM59,Mouse IgG1 κ,Biolegend,303121,1:50
CD185,BV711,CXCR5,J252D4,Mouse IgG1 κ,Biolegend,356934,1:12.5
Ki67,BV785,,B56,Mouse IgG1 κ,BD Biosciences,563756,1:50
CD45RA,FITC,,HI100,Mouse IgG2b κ,Biolegend,304105,1:100
CD3,PerCP-Cy5.5,,OKT3,Mouse IgG2a κ,Biolegend,317336,1:25
IKZF3,PE,Aiolos,16D9C97,Mouse IgG1 κ,Biolegend,371103,1:25
CD4,PE-Dazzle,,RPA-T4,Mouse IgG1 κ,Biolegend,300548,1:50
CD279,PE-Cy7,PD1,EH12.2H7,Mouse IgG1 κ,Biolegend,329918,1:25
FoxP3,AF647,,259D/C7,Mouse IgG1 κ,BD Biosciences,560889,1:10
CD69,AF700,,FN50,Mouse IgG1 κ,Biolegend,310922,1:25
CD8,APC-Cy7,,HIT3a,Mouse IgG1 κ,Biolegend,300926,1:25
CD244,BV421,,C1.7,Mouse IgG1 κ,Biolegend,329531,1:25
CD31,BV605,,WM59,Mouse IgG1 κ,Biolegend,303121,1:25
CD366,BV711,TIM3,F38-2E2,Mouse IgG1 κ,Biolegend,345023,1:25

CODEX
Target,Clone,Supplier,Catalogue (conjugated),CODEX oligo,Dilution

BCL6,K112-91,BD Biosciences,561520,79,1:25
GATA3,L50-823,Cell Marque,custom conjugation,2,1:50
CD185,D6L3C,Cell Signaling Technology,custom conjugation,69,1:100
Tbet,D6N8B,Cell Signaling Technology,custom conjugation,68,1:100
CD62L,B-8,Santa Cruz Biotechnology,custom conjugation,38,1:400
FoxP3,236A/E7,Invitrogen,14-4777-82,61,1:100
CD163,EDHu-1,Novus Biologicals,NB110-40686,59,1:50
Ki67,B56,BD Biosciences,556003,6,1:200
CD366,polyclonal,Novus Biologicals,AF2365,44,1:100
PAX5,D7H5X,Cell Signaling Technology,custom conjugation,66,1:200
CD134,Ber-ACT35,Biolegend,350002,75,1:100
IL10,polyclonal,R&D Systems,AF-217-NA,67,1:100
CD5,vC5/473 + CD5/54/F6,Novus Biologicals,NBP2-34583,25,1:50
CD206,MM0820-48L31,Abcam,custom conjugation,55,1:200
CD25,4C9,Cell Marque,custom conjugation,24,1:200
CD16,D1N9L,Cell Signaling Technology,custom conjugation,60,1:50
CD152,BSB-88,BioSB,BSB 2885 (ASR),30,1:25
CD79a,HM47,Biolegend,333502,46,1:200
CD57,HNK-1,Biolegend,359602,29,1:50
CD34,QBEnd/10,Novus Biologicals,NBP2-34713,11,1:50
CXCL13,polyclonal,Novus,AF801,41,1:200
CD21,SP186,Abcam,ab240987,15,1:100
CD7,MRQ56,Cell Marque,custom conjugation,63,1:100
Podoplanin,D2-40,Biolegend,916606,32,1:200
CD279,D4W2J,Cell Signaling Technology,custom conjugation,23,1:50
HLA-DR,EPR3692,Abcam,ab209968,65,1:100
CD223,D2G4O,Cell Signaling Technology,custom conjugation,42,1:25
CD20,rIGEL/773,Novus Biologicals,NBP2-54591,48,1:200
CD56,MRQ-42,Cell Marque,custom conjugation,58,1:100
CD45RO,UCH-L1,Santa Cruz Biotechnology,custom conjugation,5,1:50
CD278,D1K2T,Cell Signaling Technology,custom conjugation,74,1:200
CD90,EPR3132,Abcam,ab181885,57,1:150
CD4,EPR6855,Abcam,ab181724,20,1:100
CD11c,EP1347Y,Abcam,ab216655,49,1:200
CD3,MRQ-39,Cell Marque,custom conjugation,33,1:50
CD68,KP-1,BioLegend,916104,62,1:200
CD69,EPR21814,Abcam,ab234512,36,1:500
CD14,EPR3653,Abcam,ab226121,7,1:300
CD8,C8/144B,Cell Marque,custom conjugation,8,1:100
Kappa light chain,L1C1,Cell Marque,custom conjugation,70,1:100
CD45RA,HI100,Biolegend,304102,21,1:200
CD11b,EPR1344,Abcam,ab209970,28,1:200
Granzyme B,EPR20129-217,Abcam,ab219803,81,1:200
CD31,C31.3 + C31.7 + C31.10,Novus Biologicals,NBP2-47785,51,1:200
CD45,2B11+PD7/263,Novus Biologicals,NBP2-34528,56,1:200
CD38,EPR4106,Abcam,ab176886,3,1:200
CD44,IM7,Biolegend,103002,45,1:200
CD15,MMA,BD Biosciences,559045,14,1:200
Lambda light chain,Lamb14,Cell Marque,custom conjugation,26,1:200
Mast cell tryptase,AA1,Abcam,ab2378,71,1:200

Validation

CITE-seq
Target,Gene,Clone,Validation
CD10,MME,HI10a,Commercial product, tested and titrated using the corresponding PE-conjugated antibody in flow cytometry (as recommended by the manufacturer) in peripheral blood mononuclear cells
CD103,ITGAE,Ber-ACT8 ,Commercial product, tested and titrated using the corresponding PE-conjugated antibody in flow cytometry (as recommended by the manufacturer) in peripheral blood mononuclear cells
CD11b,ITGAM,ICRF44,Commercial product, tested and titrated using the corresponding PE-conjugated antibody in flow cytometry (as recommended by the manufacturer) in peripheral blood mononuclear cells
CD11c,ITGAX,S-HCL-3,Commercial product, tested and titrated using the corresponding PE-conjugated antibody in flow cytometry (as recommended by the manufacturer) in peripheral blood mononuclear cells
CD127,IL7R,A019D5,Commercial product, tested and titrated using the corresponding PE-conjugated antibody in flow cytometry (as recommended by the manufacturer) in peripheral blood mononuclear cells
CD134,TNFRSF4,Ber-ACT35,Commercial product, tested and titrated using the corresponding PE-conjugated antibody in flow cytometry (as recommended by the manufacturer) in peripheral blood mononuclear cells
CD137,TNFRSF9,4B4-1,Commercial product, tested and titrated using the corresponding PE-conjugated antibody in flow cytometry (as recommended by the manufacturer) in peripheral blood mononuclear cells
CD150,SLAMF1,A12 (7D4),Commercial product, tested and titrated using the corresponding PE-conjugated antibody in flow cytometry (as recommended by the manufacturer) in peripheral blood mononuclear cells
CD152,CTLA4,BNI3,Commercial product, tested and titrated using the corresponding PE-conjugated antibody in flow cytometry (as recommended by the manufacturer) in peripheral blood mononuclear cells
CD16,FCGR3A,3G8,Commercial product, tested and titrated using the corresponding PE-conjugated antibody in flow cytometry (as recommended by the manufacturer) in peripheral blood mononuclear cells
CD161,KLRB1,HP-3G10,Commercial product, tested and titrated using the corresponding PE-conjugated antibody in flow cytometry (as recommended by the manufacturer) in peripheral blood mononuclear cells
CD183,CXCR3,G025H7,Commercial product, tested and titrated using the corresponding PE-conjugated antibody in flow cytometry

(as recommended by the manufacturer) in peripheral blood mononuclear cells
CD184,CXCR4,12G5,Commercial product, tested and titrated using the corresponding PE-conjugated antibody in flow cytometry (as recommended by the manufacturer) in peripheral blood mononuclear cells
CD185,CXCR5,J252D4,Commercial product, tested and titrated using the corresponding PE-conjugated antibody in flow cytometry (as recommended by the manufacturer) in peripheral blood mononuclear cells
CD19,CD19,HIB19,Commercial product, tested and titrated using the corresponding PE-conjugated antibody in flow cytometry (as recommended by the manufacturer) in peripheral blood mononuclear cells
CD194,CCR4,L291H4,Commercial product, tested and titrated using the corresponding PE-conjugated antibody in flow cytometry (as recommended by the manufacturer) in peripheral blood mononuclear cells
CD195,CCR5,J418F1,Commercial product, tested and titrated using the corresponding PE-conjugated antibody in flow cytometry (as recommended by the manufacturer) in peripheral blood mononuclear cells
CD197,CCR7,G043H7,Commercial product, tested and titrated using the corresponding PE-conjugated antibody in flow cytometry (as recommended by the manufacturer) in peripheral blood mononuclear cells
CD2,CD2,TS1/8,Commercial product, tested and titrated using the corresponding PE-conjugated antibody in flow cytometry (as recommended by the manufacturer) in peripheral blood mononuclear cells
CD20,MS4A1,2H7,Commercial product, tested and titrated using the corresponding PE-conjugated antibody in flow cytometry (as recommended by the manufacturer) in peripheral blood mononuclear cells
CD21,CR2,Bu32,Commercial product, tested and titrated using the corresponding PE-conjugated antibody in flow cytometry (as recommended by the manufacturer) in peripheral blood mononuclear cells
CD22,CD22,S-HCL-1,Commercial product, tested and titrated using the corresponding PE-conjugated antibody in flow cytometry (as recommended by the manufacturer) in peripheral blood mononuclear cells
CD223,LAG3,11C3C65,Commercial product, tested and titrated using the corresponding PE-conjugated antibody in flow cytometry (as recommended by the manufacturer) in peripheral blood mononuclear cells
CD23,FCER2,EBVCS-5,Commercial product, tested and titrated using the corresponding PE-conjugated antibody in flow cytometry (as recommended by the manufacturer) in peripheral blood mononuclear cells
CD24,CD24,ML5,Commercial product, tested and titrated using the corresponding PE-conjugated antibody in flow cytometry (as recommended by the manufacturer) in peripheral blood mononuclear cells
CD244,CD244,C1.7,Commercial product, tested and titrated using the corresponding PE-conjugated antibody in flow cytometry (as recommended by the manufacturer) in peripheral blood mononuclear cells
CD25,IL2RA,BC96,Commercial product, tested and titrated using the corresponding PE-conjugated antibody in flow cytometry (as recommended by the manufacturer) in peripheral blood mononuclear cells
CD27,CD27,O323,Commercial product, tested and titrated using the corresponding PE-conjugated antibody in flow cytometry (as recommended by the manufacturer) in peripheral blood mononuclear cells
CD273,PDCD1LG2,24F.10C12,Commercial product, tested and titrated using the corresponding PE-conjugated antibody in flow cytometry (as recommended by the manufacturer) in peripheral blood mononuclear cells
CD274,PDCD1LG1,29E.2A3,Commercial product, tested and titrated using the corresponding PE-conjugated antibody in flow cytometry (as recommended by the manufacturer) in peripheral blood mononuclear cells
CD278,ICOS,C398.4A,Commercial product, tested and titrated using the corresponding PE-conjugated antibody in flow cytometry (as recommended by the manufacturer) in peripheral blood mononuclear cells
CD279,PDCD1,EH12.2H7,Commercial product, tested and titrated using the corresponding PE-conjugated antibody in flow cytometry (as recommended by the manufacturer) in peripheral blood mononuclear cells
CD28,CD28,CD28.2,Commercial product, tested and titrated using the corresponding PE-conjugated antibody in flow cytometry (as recommended by the manufacturer) in peripheral blood mononuclear cells
CD29,ITGB1,TS2/16,Commercial product, tested and titrated using the corresponding PE-conjugated antibody in flow cytometry (as recommended by the manufacturer) in peripheral blood mononuclear cells
CD3,CD3E,UCHT1,Commercial product, tested and titrated using the corresponding PE-conjugated antibody in flow cytometry (as recommended by the manufacturer) in peripheral blood mononuclear cells
CD31,PECAM1,WM59,Commercial product, tested and titrated using the corresponding PE-conjugated antibody in flow cytometry (as recommended by the manufacturer) in peripheral blood mononuclear cells
CD32,FCGR2A,FUN-2,Commercial product, tested and titrated using the corresponding PE-conjugated antibody in flow cytometry (as recommended by the manufacturer) in peripheral blood mononuclear cells
CD357,TNFRSF18,108-17,Commercial product, tested and titrated using the corresponding PE-conjugated antibody in flow cytometry (as recommended by the manufacturer) in peripheral blood mononuclear cells
CD366,HAVCR2,F38-2E2,Commercial product, tested and titrated using the corresponding PE-conjugated antibody in flow cytometry (as recommended by the manufacturer) in peripheral blood mononuclear cells
CD38,CD38,HIT2,Commercial product, tested and titrated using the corresponding PE-conjugated antibody in flow cytometry (as recommended by the manufacturer) in peripheral blood mononuclear cells
CD39,ENTPD1,A1,Commercial product, tested and titrated using the corresponding PE-conjugated antibody in flow cytometry (as recommended by the manufacturer) in peripheral blood mononuclear cells
CD4,CD4,RPA-T4,Commercial product, tested and titrated using the corresponding PE-conjugated antibody in flow cytometry (as recommended by the manufacturer) in peripheral blood mononuclear cells
CD43,SPN,CD43-10G7,Commercial product, tested and titrated using the corresponding PE-conjugated antibody in flow cytometry (as recommended by the manufacturer) in peripheral blood mononuclear cells
CD44,CD44,IM7,Commercial product, tested and titrated using the corresponding PE-conjugated antibody in flow cytometry (as recommended by the manufacturer) in peripheral blood mononuclear cells
CD45,PTPRC,HI30,Commercial product, tested and titrated using the corresponding PE-conjugated antibody in flow cytometry (as recommended by the manufacturer) in peripheral blood mononuclear cells
CD45RA,PTPRC,HI100,Commercial product, tested and titrated using the corresponding PE-conjugated antibody in flow cytometry (as recommended by the manufacturer) in peripheral blood mononuclear cells
CD45RO,PTPRC,UCHL1,Commercial product, tested and titrated using the corresponding PE-conjugated antibody in flow cytometry (as recommended by the manufacturer) in peripheral blood mononuclear cells
CD47,CD47,CC2C6,Commercial product, tested and titrated using the corresponding PE-conjugated antibody in flow cytometry (as recommended by the manufacturer) in peripheral blood mononuclear cells
CD48,CD48,BJ40,Commercial product, tested and titrated using the corresponding PE-conjugated antibody in flow cytometry (as recommended by the manufacturer) in peripheral blood mononuclear cells
CD5,CD5,UCHT2,Commercial product, tested and titrated using the corresponding PE-conjugated antibody in flow cytometry (as

recommended by the manufacturer) in peripheral blood mononuclear cells
CD56,NCAM1,QA17A16,Commercial product, tested and titrated using the corresponding PE-conjugated antibody in flow cytometry (as recommended by the manufacturer) in peripheral blood mononuclear cells
CD57,B3GAT1,QA17A04,Commercial product, tested and titrated using the corresponding PE-conjugated antibody in flow cytometry (as recommended by the manufacturer) in peripheral blood mononuclear cells
CD62L,SELL,DREG-56,Commercial product, tested and titrated using the corresponding PE-conjugated antibody in flow cytometry (as recommended by the manufacturer) in peripheral blood mononuclear cells
CD69,CD69,FN50,Commercial product, tested and titrated using the corresponding PE-conjugated antibody in flow cytometry (as recommended by the manufacturer) in peripheral blood mononuclear cells
CD7,CD7,CD7-6B7,Commercial product, tested and titrated using the corresponding PE-conjugated antibody in flow cytometry (as recommended by the manufacturer) in peripheral blood mononuclear cells
CD70,CD70,113-16,Commercial product, tested and titrated using the corresponding PE-conjugated antibody in flow cytometry (as recommended by the manufacturer) in peripheral blood mononuclear cells
CD73,NT5E,AD2,Commercial product, tested and titrated using the corresponding PE-conjugated antibody in flow cytometry (as recommended by the manufacturer) in peripheral blood mononuclear cells
CD79b,CD79B,CB3-1,Commercial product, tested and titrated using the corresponding PE-conjugated antibody in flow cytometry (as recommended by the manufacturer) in peripheral blood mononuclear cells
CD86,CD86,IT2.2,Commercial product, tested and titrated using the corresponding PE-conjugated antibody in flow cytometry (as recommended by the manufacturer) in peripheral blood mononuclear cells
CD8a,CD8A,RPA-T8,Commercial product, tested and titrated using the corresponding PE-conjugated antibody in flow cytometry (as recommended by the manufacturer) in peripheral blood mononuclear cells
CD95,FAS,DX2,Commercial product, tested and titrated using the corresponding PE-conjugated antibody in flow cytometry (as recommended by the manufacturer) in peripheral blood mononuclear cells
Isotype Ctrl,,MOPC-21,Commercial product, tested and titrated using the corresponding PE-conjugated antibody in flow cytometry (as recommended by the manufacturer) in peripheral blood mononuclear cells
Isotype Ctrl,,HTK888,Commercial product, tested and titrated using the corresponding PE-conjugated antibody in flow cytometry (as recommended by the manufacturer) in peripheral blood mononuclear cells
Isotype Ctrl,,MPC-11,Commercial product, tested and titrated using the corresponding PE-conjugated antibody in flow cytometry (as recommended by the manufacturer) in peripheral blood mononuclear cells
Isotype Ctrl,,RTK4530,Commercial product, tested and titrated using the corresponding PE-conjugated antibody in flow cytometry (as recommended by the manufacturer) in peripheral blood mononuclear cells
Isotype Ctrl,,MOPC-173,Commercial product, tested and titrated using the corresponding PE-conjugated antibody in flow cytometry (as recommended by the manufacturer) in peripheral blood mononuclear cells
Kappa,IGKC,MHK-49 ,Commercial product, tested and titrated using the corresponding PE-conjugated antibody in flow cytometry (as recommended by the manufacturer) in peripheral blood mononuclear cells
KLRG1,KLRG1,SA231A2,Commercial product, tested and titrated using the corresponding PE-conjugated antibody in flow cytometry (as recommended by the manufacturer) in peripheral blood mononuclear cells
Lambda,IGLC2,MHL-38 ,Commercial product, tested and titrated using the corresponding PE-conjugated antibody in flow cytometry (as recommended by the manufacturer) in peripheral blood mononuclear cells
TIGIT,TIGIT,A15153G,Commercial product, tested and titrated using the corresponding PE-conjugated antibody in flow cytometry (as recommended by the manufacturer) in peripheral blood mononuclear cells

Flow Cytometry
Target,Conjugate,Clone,
CD25,BV421,BC96,Commercial product, routinely tested and extenisvely titrated in peripheral blood mononuclear cells
CD278,BV605,WM59,Commercial product, routinely tested and extenisvely titrated in peripheral blood mononuclear cells
CD185,BV711,J252D4,Commercial product, routinely tested and extenisvely titrated in peripheral blood mononuclear cells
Ki67,BV785,B56,Commercial product, routinely tested and extenisvely titrated in peripheral blood mononuclear cells
CD45RA,FITC,HI100,Commercial product, routinely tested and extenisvely titrated in peripheral blood mononuclear cells
CD3,PerCP-Cy5.5,OKT3,Commercial product, routinely tested and extenisvely titrated in peripheral blood mononuclear cells
IKZF3,PE,16D9C97,Commercial product, routinely tested and extenisvely titrated in peripheral blood mononuclear cells
CD4,PE-Dazzle,RPA-T4,Commercial product, routinely tested and extenisvely titrated in peripheral blood mononuclear cells
CD279,PE-Cy7,EH12.2H7,Commercial product, routinely tested and extenisvely titrated in peripheral blood mononuclear cells
FoxP3,AF647,259D/C7,Commercial product, routinely tested and extenisvely titrated in peripheral blood mononuclear cells
CD69,AF700,FN50,Commercial product, routinely tested and extenisvely titrated in peripheral blood mononuclear cells
CD8,APC-Cy7,HIT3a,Commercial product, routinely tested and extenisvely titrated in peripheral blood mononuclear cells
CD244,BV421,C1.7,Commercial product, routinely tested and extenisvely titrated in peripheral blood mononuclear cells
CD31,BV605,WM59,Commercial product, routinely tested and extenisvely titrated in peripheral blood mononuclear cells
CD366,BV711,F38-2E2,Commercial product, routinely tested and extenisvely titrated in peripheral blood mononuclear cells

CODEX
Target,Clone,CODEX oligo,Validation
BCL6,K112-91,79,Commercial product, extensive titration and validation in tonsils and tumor-free lymph nodes, reviewed by two independent board-certified hemato-pathologists
GATA3,L50-823,2,Custom conjugation of commercially available unconjugated antibody, extensive titration and validation in tonsils and tumor-free lymph nodes, reviewed by two independent board-certified hemato-pathologists
CD185,D6L3C,69,Custom conjugation of commercially available unconjugated antibody, extensive titration and validation in tonsils and tumor-free lymph nodes, reviewed by two independent board-certified hemato-pathologists
Tbet,D6N8B,68,Custom conjugation of commercially available unconjugated antibody, extensive titration and validation in tonsils and tumor-free lymph nodes, reviewed by two independent board-certified hemato-pathologists
CD62L,B-8,38,Custom conjugation of commercially available unconjugated antibody, extensive titration and validation in tonsils and tumor-free lymph nodes, reviewed by two independent board-certified hemato-pathologists
FoxP3,236A/E7,61,Commercial product, extensive titration and validation in tonsils and tumor-free lymph nodes, reviewed by two independent board-certified hemato-pathologists
CD163,EDHu-1,59,Commercial product, extensive titration and validation in tonsils and tumor-free lymph nodes, reviewed by two independent board-certified hemato-pathologists

Ki67,B56,6,Commercial product, extensive titration and validation in tonsils and tumor-free lymph nodes, reviewed by two independent board-certified hemato-pathologists
CD366,polyclonal,44,Commercial product, extensive titration and validation in tonsils and tumor-free lymph nodes, reviewed by two independent board-certified hemato-pathologists
PAX5,D7H5X,66,Custom conjugation of commercially available unconjugated antibody, extensive titration and validation in tonsils and tumor-free lymph nodes, reviewed by two independent board-certified hemato-pathologists
CD134,Ber-ACT35,75,Commercial product, extensive titration and validation in tonsils and tumor-free lymph nodes, reviewed by two independent board-certified hemato-pathologists
IL10,polyclonal,67,Commercial product, extensive titration and validation in tonsils and tumor-free lymph nodes, reviewed by two independent board-certified hemato-pathologists
CD5,vC5/473 + CD5/54/F6,25,Commercial product, extensive titration and validation in tonsils and tumor-free lymph nodes, reviewed by two independent board-certified hemato-pathologists
CD206,MM0820-48L31,55,Custom conjugation of commercially available unconjugated antibody, extensive titration and validation in tonsils and tumor-free lymph nodes, reviewed by two independent board-certified hemato-pathologists
CD25,4C9,24,Custom conjugation of commercially available unconjugated antibody, extensive titration and validation in tonsils and tumor-free lymph nodes, reviewed by two independent board-certified hemato-pathologists
CD16,D1N9L,60,Custom conjugation of commercially available unconjugated antibody, extensive titration and validation in tonsils and tumor-free lymph nodes, reviewed by two independent board-certified hemato-pathologists
CD152,BSB-88,30,Commercial product, extensive titration and validation in tonsils and tumor-free lymph nodes, reviewed by two independent board-certified hemato-pathologists
CD79a,HM47,46,Commercial product, extensive titration and validation in tonsils and tumor-free lymph nodes, reviewed by two independent board-certified hemato-pathologists
CD57,HNK-1,29,Commercial product, extensive titration and validation in tonsils and tumor-free lymph nodes, reviewed by two independent board-certified hemato-pathologists
CD34,QBEnd/10,11,Commercial product, extensive titration and validation in tonsils and tumor-free lymph nodes, reviewed by two independent board-certified hemato-pathologists
CXCL13,polyclonal,41,Commercial product, extensive titration and validation in tonsils and tumor-free lymph nodes, reviewed by two independent board-certified hemato-pathologists
CD21,SP186,15,Commercial product, extensive titration and validation in tonsils and tumor-free lymph nodes, reviewed by two independent board-certified hemato-pathologists
CD7,MRQ56,63,Custom conjugation of commercially available unconjugated antibody, extensive titration and validation in tonsils and tumor-free lymph nodes, reviewed by two independent board-certified hemato-pathologists
Podoplanin,D2-40,32,Commercial product, extensive titration and validation in tonsils and tumor-free lymph nodes, reviewed by two independent board-certified hemato-pathologists
CD279,D4W2J,23,Custom conjugation of commercially available unconjugated antibody, extensive titration and validation in tonsils and tumor-free lymph nodes, reviewed by two independent board-certified hemato-pathologists
HLA-DR,EPR3692,65,Commercial product, extensive titration and validation in tonsils and tumor-free lymph nodes, reviewed by two independent board-certified hemato-pathologists
CD223,D2G4O,42,Custom conjugation of commercially available unconjugated antibody, extensive titration and validation in tonsils and tumor-free lymph nodes, reviewed by two independent board-certified hemato-pathologists
CD20,rIGEL/773,48,Commercial product, extensive titration and validation in tonsils and tumor-free lymph nodes, reviewed by two independent board-certified hemato-pathologists
CD56,MRQ-42,58,Custom conjugation of commercially available unconjugated antibody, extensive titration and validation in tonsils and tumor-free lymph nodes, reviewed by two independent board-certified hemato-pathologists
CD45RO,UCH-L1,5,Custom conjugation of commercially available unconjugated antibody, extensive titration and validation in tonsils and tumor-free lymph nodes, reviewed by two independent board-certified hemato-pathologists
CD278,D1K2T,74,Custom conjugation of commercially available unconjugated antibody, extensive titration and validation in tonsils and tumor-free lymph nodes, reviewed by two independent board-certified hemato-pathologists
CD90,EPR3132,57,Commercial product, extensive titration and validation in tonsils and tumor-free lymph nodes, reviewed by two independent board-certified hemato-pathologists
CD4,EPR6855,20,Commercial product, extensive titration and validation in tonsils and tumor-free lymph nodes, reviewed by two independent board-certified hemato-pathologists
CD11c,EP1347Y,49,Commercial product, extensive titration and validation in tonsils and tumor-free lymph nodes, reviewed by two independent board-certified hemato-pathologists
CD3,MRQ-39,33,Custom conjugation of commercially available unconjugated antibody, extensive titration and validation in tonsils and tumor-free lymph nodes, reviewed by two independent board-certified hemato-pathologists
CD68,KP-1,62,Commercial product, extensive titration and validation in tonsils and tumor-free lymph nodes, reviewed by two independent board-certified hemato-pathologists
CD69,EPR21814,36,Commercial product, extensive titration and validation in tonsils and tumor-free lymph nodes, reviewed by two independent board-certified hemato-pathologists
CD14,EPR3653,7,Commercial product, extensive titration and validation in tonsils and tumor-free lymph nodes, reviewed by two independent board-certified hemato-pathologists
CD8,C8/144B,8,Custom conjugation of commercially available unconjugated antibody, extensive titration and validation in tonsils and tumor-free lymph nodes, reviewed by two independent board-certified hemato-pathologists
Kappa light chain,L1C1,70,Custom conjugation of commercially available unconjugated antibody, extensive titration and validation in tonsils and tumor-free lymph nodes, reviewed by two independent board-certified hemato-pathologists
CD45RA,HI100,21,Commercial product, extensive titration and validation in tonsils and tumor-free lymph nodes, reviewed by two independent board-certified hemato-pathologists
CD11b,EPR1344,28,Commercial product, extensive titration and validation in tonsils and tumor-free lymph nodes, reviewed by two independent board-certified hemato-pathologists
Granzyme B,EPR20129-217,81,Commercial product, extensive titration and validation in tonsils and tumor-free lymph nodes, reviewed by two independent board-certified hemato-pathologists
CD31,C31.3 + C31.7 + C31.10,51,Commercial product, extensive titration and validation in tonsils and tumor-free lymph nodes, reviewed by two independent board-certified hemato-pathologists
CD45,2B11+PD7/263,56,Commercial product, extensive titration and validation in tonsils and tumor-free lymph nodes, reviewed by two independent board-certified hemato-pathologists

CD38,EPR4106,3,Commercial product, extensive titration and validation in tonsils and tumor-free lymph nodes, reviewed by two independent board-certified hemato-pathologists
CD44,IM7,45,Commercial product, extensive titration and validation in tonsils and tumor-free lymph nodes, reviewed by two independent board-certified hemato-pathologists
CD15,MMA,14,Commercial product, extensive titration and validation in tonsils and tumor-free lymph nodes, reviewed by two independent board-certified hemato-pathologists
Lambda light chain,Lamb14,26,Custom conjugation of commercially available unconjugated antibody, extensive titration and validation in tonsils and tumor-free lymph nodes, reviewed by two independent board-certified hemato-pathologists
Mast cell tryptase,AA1,71,Commercial product, extensive titration and validation in tonsils and tumor-free lymph nodes, reviewed by two independent board-certified hemato-pathologists

Flow Cytometry

Plots

Confirm that:

- ☒ The axis labels state the marker and fluorochrome used (e.g. CD4-FITC).
- ☒ The axis scales are clearly visible. Include numbers along axes only for bottom left plot of group (a 'group' is an analysis of identical markers).
- ☒ All plots are contour plots with outliers or pseudocolor plots.
- ☒ A numerical value for number of cells or percentage (with statistics) is provided.

Methodology

Sample preparation	LN-derived cells were thawed, washed and stained for viability using a fixable viability dye e506 (Thermo Fisher Scientific) and for different surface markers depending on the experimental set-up. For subsequent intracellular staining, cells were fixed and permeabilized with the intracellular fixation/permeabilization buffer set (Thermo Fisher Scientific) and stained. Then, cells were analyzed.
Instrument	LSR Fortessa (BD Biosciences)
Software	FACSDiva (BD Biosciences, version 8), FlowJo (v10.8.0)
Cell population abundance	Not relevant. Sorting was not applied.
Gating strategy	Gating strategies are summarized in detail in Supplementary Figure 2 and 3.

☒ Tick this box to confirm that a figure exemplifying the gating strategy is provided in the Supplementary Information.
